# Sex Differences in Psychopathology Following Potentially Traumatic Experiences

**DOI:** 10.1001/jamanetworkopen.2024.0201

**Published:** 2024-02-22

**Authors:** Yasmin B. Kofman, Sophie Selbe, Peter Szentkúti, Erzsébet Horváth-Puhó, Anthony J. Rosellini, Timothy L. Lash, Paula P. Schnurr, Henrik Toft Sørensen, Sandro Galea, Jaimie L. Gradus, Jennifer A. Sumner

**Affiliations:** 1 Department of Psychology, University of California, Los Angeles; 2 Department of Epidemiology, Boston University School of Public Health, Boston, Massachusetts; 3 Department of Clinical Epidemiology, Aarhus University Hospital and Aarhus University, Aarhus, Denmark; 4 Department of Epidemiology, Rollins School of Public Health, Emory University, Atlanta, Georgia; 5 National Center for PTSD Executive Division, White River Junction, Vermont; 6 Geisel School of Medicine at Dartmouth, Hanover, New Hampshire; 7 Department of Psychiatry, Boston University School of Medicine, Boston, Massachusetts; 8 Department of Psychological and Brain Sciences, Boston University, Boston, Massachusetts

## Abstract

**Importance:**

Various psychopathology may follow trauma; however, sex differences in these ranging manifestations of posttraumatic psychopathology remain understudied.

**Objective:**

To investigate sex-specific incidence of posttraumatic psychopathology.

**Design, Setting, and Participants:**

This population-based cohort study of Danish national health registries included a cohort of individuals who experienced a potentially traumatic event (PTE) from 1994 to 2016. Individuals were further categorized by presence of any pretrauma psychopathology. A comparison group of individuals who experienced a nontraumatic stressor (nonsuicide death of a first-degree relative) was examined as a reference cohort.

**Exposures:**

At least 1 of 8 PTEs (eg, physical assault, transportation accident) derived through health registry *International Statistical Classification of Diseases and Related Health Problems, Tenth Revision* (*ICD-10*) codes, with additional qualifiers to improve classification accuracy.

**Main Outcomes and Measures:**

Incidence of 9 categories of *ICD-10* psychiatric disorders recorded in registries within 5 years of PTEs. The standardized morbidity ratios (SMRs) for psychopathology outcomes were also calculated to compare individuals experiencing PTEs with those experiencing a nontraumatic stressor.

**Results:**

This study included 1 398 026 individuals who had been exposed to trauma (475 280 males [34.0%]; 922 750 females [66.0%]). The group of males who had been exposed to trauma were evenly distributed across age, while most females in the trauma-exposed group were aged 16 to 39 years (592 385 [64.2%]). Males and females were equally distributed across income quartiles and predominantly single. Following PTEs, the most common diagnosis was substance use disorders for males (35 160 [7.4%]) and depressive disorders for females (29 255 [3.2%]); incidence proportions for these and other disorders were higher among males and females with any pretrauma psychopathology. Certain PTEs had elevated onset of various psychiatric disorders and some sex differences emerged. Following physical assault, associations were found with schizophrenia or psychotic disorders for males (SMR, 17.5; 95% CI, 15.9-19.3) and adult personality disorders for females (SMR, 16.3; 95% CI, 14.6-18.3). For noninterpersonal PTEs, males had larger SMRs for substance use, schizophrenia or psychotic disorders, and adult personality disorders (SMR, 43.4; 95% CI, 41.9-45.0), and females had larger SMRs for depressive disorders (SMR, 19.0; 95% CI, 18.6-19.4). Sex differences were also observed, particularly when considering pretrauma psychopathology. For example, among interpersonal PTEs, males were most likely to develop substance use disorders after physical assault, whereas females were more likely to develop various disorders, with stronger associations seen for females without pretrauma psychiatric diagnoses. Among noninterpersonal PTEs, exposure to toxic substance showed robust associations with psychopathology, particularly in those without pretrauma psychopathology, with sex-specific differences across psychiatric categories.

**Conclusions and Relevance:**

Mental disorders after trauma were wide-ranging for males and females, and sex differences in patterns of posttraumatic psychopathology were more pronounced when accounting for pretrauma psychopathology. Findings provide new insights for sex-relevant PTEs and their mental health consequences. It also outlines future directions for advancing understanding of a constellation of posttraumatic psychopathology in males and females.

## Key Points

**Question:**

What is the sex-specific incidence of various forms of posttraumatic psychopathology in a population-based cohort?

**Findings:**

In this cohort study of more than 1.3 million individuals, patterns of 5-year posttraumatic psychopathology incidence showed substance use disorders were most common for males and depressive disorders were the most common for females. Sex-based differences in associations of potentially traumatic events with psychopathology were more pronounced when accounting for pretrauma psychiatric disorders.

**Meaning:**

These findings suggest that mental health consequences of trauma in males and females are sex-specific and wide-ranging and may provide new insights for sex-relevant potentially traumatic experiences and their mental health consequences.

## Teaser

This cohort study examines the sex-specific incidence of posttraumatic psychopathology among Danish individuals who experienced a potentially traumatic event from 1994 to 2016.

## Introduction

Trauma can have lasting adverse effects on mental health.^1,2^ Although most studies have focused on posttraumatic stress disorder (PTSD), as it is contingent upon trauma,^3^ growing research has linked potentially traumatic events (PTEs) to multiple mental health sequelae.^4,5,6,7,8^ However, despite sex differences in PTEs and prevalence of PTSD,^2,9^ research on sex differences in varied manifestations of posttraumatic psychopathology remains limited. This is an important gap to address given the heterogeneous nature of posttraumatic psychopathology and established sex differences in psychiatric disorders in the nontrauma literature.^10^

We previously harnessed Danish national health registries to identify a population-based, trauma-exposed cohort and showed that PTEs were associated with several psychiatric disorder categories, including—but not limited to—PTSD and other stress-related disorders.^11^ The current study extends this work by investigating sex differences in incident posttraumatic psychopathology, defined according to 9 diagnostic categories that onset within 5 years following PTEs. We also examined sex-specific patterns in associations between PTEs and subsequent psychopathology in comparison to individuals experiencing a nontraumatic stressor. To comprehensively consider patterns of posttraumatic psychopathology in males and females, analyses were conducted in the full cohort and separately in individuals with and without recorded psychiatric diagnoses before PTEs. Although we were interested in understanding overall manifestations of psychiatric disorders after PTEs, as this sheds light on the posttraumatic psychiatric milieu that can inform intervention efforts after trauma, we considered the potential influence of pretrauma psychiatric disorders on posttraumatic psychopathology in males and females.

## Methods

### Study Population

Our source population was all 7 420 888 residents of Danish from 1994 to 2016. Denmark residents receive medical care through a tax-funded system, with health care encounters and social variables documented in national registries.^12^ Each resident has a unique identifier, used to merge data across registries. Harnessing these registries, we identified 1 398 026 individuals experiencing at least 1 of 8 PTEs from 1994 to 2016 (described in Gradus et al^11^). Individuals entered the cohort upon their first-recorded PTE within the study period. The Danish Data Protection Agency approved this research. This cohort study followed the Strengthening the Reporting of Observational Studies in Epidemiology (STROBE) reporting guideline.^13^ Informed consent was waived because data were deidentified.

### Data Sources

Several registries contributed data for this study. Demographic data were from the Danish Civil Registration System^14,15^ and Income Statistics Register.^16^ Psychiatric diagnoses were from the Danish Psychiatric Central Research Register (DPCRR) and supplemented with psychiatric diagnoses recorded in the Danish National Patient Registry (DNPR)^17,18^; nonpsychiatric discharge diagnoses were also collected from the DNPR. Pregnancy-related trauma data were from the Danish Medical Birth Register.^19^ Death data were from the Cause of Death Registry.^20^ Registry details, including validity information, are presented in eMethods in Supplement 1.

### Variable Definitions

#### Patient Demographics and PTE Categories

Demographics included age, income quartiles, and marital status (eMethods in Supplement 1). Nine PTE categories were identified using *International Statistical Classification of Diseases and Related Health Problems, Tenth Revision* (*ICD-10*) code-based discharge diagnoses in the DNPR and DPCRR for fire or explosion, transportation accident, exposure to toxic substance, medical complications or injury, traumatic brain injury (TBI), physical assault, pregnancy-related trauma, and suicide death of a family member (eTable 1 and eMethods in Supplement 1). To capture more serious events consistent with PTEs, hospitalization of 2 days was required for transportation accident, exposure to toxic substance, and medical complications or injury and 3 days for pregnancy-related trauma (except stillbirth).^11^ If individuals experienced more than 1 event on the same day, they were coded in a multiple PTEs category. PTE categories were discrete; individuals in the cohort were classified once for first-recorded PTE.

#### Psychiatric Disorders

Nine psychiatric disorder categories were determined using *ICD-10* codes in the DPCRR and DNPR. The categories included (1) organic disorders (F00-F09); (2) substance use disorders (F10-F19); (3) schizophrenia or psychotic disorders (F20-F29); (4) manic episode or bipolar disorders (F30-F31 and F34-F39); (5) depressive disorders (F32-F33); (6) neurotic or somatoform disorders (F40-F42 and F44-F48); (7) stress-related disorders (F43); (8) physiological disorders (F50-F59); and (9) adult personality disorders (F60-F69).^9,21^ For each category, we identified incident psychiatric disorders, defined as first-recorded diagnosis within 5 years of PTEs, as in prior research.^11^ Incidence was captured for all possible disorder categories.

### Statistical Analysis

Sex-stratified demographics were examined overall and within each PTE category. Sex-stratified 5-year incidence of posttraumatic psychiatric disorders overall and within each PTE category were calculated (number of new diagnoses divided by number of individuals at risk).

Standardized morbidity ratios (SMRs), ratios of observed to expected cases, stratified by sex, were calculated to measure associations between PTEs and psychopathology relative to a nontraumatic stressor. For trauma-exposed males and females, expected numbers of cases within psychiatric disorder categories were calculated by applying outcome rates, stratified by 5-year age groups and 5-year calendar periods for a group that experienced a nontraumatic stressor (nonsuicide death of first-degree relative) within the study period (423 270 individuals). We chose this comparison to establish whether PTEs were associated with psychopathology beyond that following a nontraumatic stressor. We calculated 95% CIs assuming observed numbers of psychiatric diagnoses followed a Poisson distribution, presenting results for interpersonal PTEs and noninterpersonal PTEs (eMethods in Supplement 1).

We repeated these analyses, stratifying by presence of any documented psychiatric diagnosis before PTE (pretrauma psychopathology), as in prior research.^22^ Given variability in codes used to define the exposure to toxic substance PTE, we also estimated SMRs separately in individuals exposed to substances with abuse or overdose potential vs other substances with less potential for abuse or overdose (eMethods and eResults in Supplement 1). Final analyses were conducted in November 2023 using SAS version 9.4 (SAS Institute). Statistical significance was set at *P* <.05.

## Results

### Full Cohort Results

This study included 1 398 026 individuals who had been exposed to trauma (475 280 males [34.0%]; 922 750 females [66.0%]). Demographics by PTEs are presented in Table 1 (males) and Table 2 (females). Males who were exposed to trauma were evenly distributed across age and income groups and most were single (250 455 [52.7%]). Most trauma-exposed females were aged 16 to 39 years (592 385 [64.2%]), evenly distributed across income quartiles, and single. Regarding PTEs, males and females commonly experienced TBI (171 200 of 475 280 males [36.0%]; 125 200 of 922 750 females [13.6%]) and medical complications or injury (145 320 of 475 280 males [30.6%]; 150 995 of 922 750 females [16.4%]), although more frequently for males. For most PTEs, males had greater exposure than females, aside from pregnancy-related trauma (512 820 of 922 750 females [55.6%]; 3685 of 475 280 males with an affected partner [0.8%]).

**Table 1.** Demographic Characteristics of Males in the Danish Health Registry Trauma Cohort and Comparison Cohort

Characteristics	Individuals, No. (%)										
	Comparison cohort	Trauma cohort	Fire or explosion	Transportation accident	Exposure to toxic substance	Medical complications or injury	TBI	Physical assault	Pregnancy-related trauma^a^	Suicide of family member	Multiple traumatic events^b^
Total males, No.	148 650	475 280	67 715	14 360	46 750	145 320	171 200	16 770	3685	3875	5600
Age, y											
<16	16 625 (11.2)	101 355 (21.3)	25 340 (37.4)	2515 (17.5)	6415 (13.7)	11 890 (8.2)	50 395 (29.4)	1625 (9.7)	<10^c^	2115 (54.6)	1060 (18.9)
16-39	7580 (5.1)	145 755 (30.7)	25 295 (37.4)	3985 (27.7)	15 035 (32.2)	25 195 (17.3)	59 410 (34.7)	11 485 (68.5)	3010 (81.7)	440 (11.4)	1900 (33.9)
40-59	19 180 (12.9)	101 485 (21.4)	12 945 (19.1)	4120 (28.7)	12 995 (27.8)	34 660 (23.8)	30 860 (18.0)	3120 (18.6)	665 (18.0)	690 (17.8)	1430 (25.5)
≥60	105 265 (70.8)	126 680 (26.7)	4135 (6.1)	3740 (26.0)	12 305 (26.3)	73 580 (50.6)	30 540 (17.8)	540 (3.2)	10 (0.3)	630 (16.2)	1210 (21.6)
Income quartile											
<1 (lowest)	38 590 (26.0)	93 410 (19.7)	10 270 (15.2)	3200 (22.3)	9555 (20.4)	26 210 (18.0)	34 840 (20.4)	6935 (41.4)	350 (9.5)	730 (18.9)	1315 (23.4)
1 to <2	42.490 (28.6)	107 970 (22.7)	8470 (12.5)	2945 (20.5)	14 300 (30.6)	42 715 (29.4)	34 240 (20.0)	3285(19.6)	530 (14.4)	270 (7.0)	1215 (21.7)
2 to <3	22 665 (15.2)	74 425 (15.7)	8745 (12.9)	2425 (16.9)	8290 (17.7)	27 145 (18.7)	23 570 (13.8)	2275 (13.6)	890 (24.1)	250 (6.4)	835 (14.9)
≥3 (highest)	30 410 (20.5)	103 175 (21.7)	15 850(23.4)	3425 (24.1)	8410 (18.0)	37 650 (25.9)	30 975 (18.1)	2975 (17.7)	1880 (51.0)	725 (18.7)	1250 (22.4)
Child^d^	12 835 (8.6)	89 945 (18.9)	23 465 (34.7)	2100 (14.6)	5635 (12.1)	10 550 (7.3)	44 740 (26.1)	780 (4.6)	<10^c^	1785 (46.1)	890 (15.9)
Missing	1655 (1.1)	6355 (1.3)	915 (1.4)	225 (1.6)	560 (1.2)	1055 (0.7)	2825 (1.7)	525 (3.1)	40 (1.0)	110 (2.9)	100 (1.8)
Marital status											
Married or registered partner	125 095 (84.2)	154 170 (32.4)	15 530 (22.9)	5145 (35.8)	14 630 (31.3)	73 405 (50.5)	37 885 (22.1)	2625 (15.7)	1965 (53.4)	1350 (34.8)	1635 (29.2)
Single	22 305 (15.0)	250 455 (52.7)	46 875 (69.2)	7240 (50.4)	23 085 (49.4)	42 755 (29.4)	110 690 (64.7)	12 690 (75.7)	1530 (41.5)	2460 (63.5)	3130 (55.9)
Divorced	825 (0.6)	42 575 (9.0)	3560 (5.3)	1325 (9.2)	6145 (13.1)	15 875 (10.9)	13 725 (8.0)	1190 (7.1)	180 (4.8)	30 (0.8)	545 (9.7)
Widowed	45 (<0.1)	23 200 (4.9)	640 (0.9)	585 (4.1)	2660 (5.7)	12 425 (8.6)	6595 (3.9)	70 (0.4)	<10^c^	<10^c^	220 (3.9)
Unknown	380 (0.3)	4875 (1.0)	1115 (1.6)	65 (0.5)	235 (0.5)	855 (0.6)	2305 (1.3)	190 (1.1)	<10^c^	35 (0)	70 (1.3)

Abbreviation: TBI, traumatic brain injury.

^a^ Males included in the pregnancy-related trauma category are those who experienced the stillbirth of a child.

^b^ Indicates multiple traumas on the day of the first-recorded trauma.

^c^ Cells with a sample size of 5 or less and cells in which a value presented could result in the calculation of a sample size of less than 5 in a complementary cell are presented as less than 10.

^d^ Persons under the age of 14 with no income data.

**Table 2.** Demographic Characteristics of Females in the Danish Health Registry Trauma Cohort and Comparison Cohort^**a**^

Characteristics	Individuals, No. (%)										
	Comparison cohort	Trauma cohort	Fire or explosion	Transportation accident	Exposure to toxic substance	Medical complications or injury	TBI	Physical assault	Pregnancy-related trauma	Suicide of family member	Multiple traumatic events^b^
Total females, No.	271 895	922 750	46 280	9385	62 125	150 995	125 200	6845	512 820	5715	3375
Age, y											
<16	13 325 (4.9)	81 610 (8.8)	18 530 (40.0)	1625 (17.3)	9870 (15.9)	8740 (5.8)	39 080 (31.2)	765 (11.2)	225 (<0.1)	2050 (35.9)	725 (21.4)
16-39	5353 (2.0)	592 385 (64.2)	15 080 (32.6)	1420 (15.1)	20 695 (33.3)	19 470 (12.9)	33 595 (26.8)	4200 (61.4)	496 225 (96.8)	730 (12.8)	965 (28.6)
40-59	39 100 (14.4)	98 500 (10.7)	8295 (17.9)	1750 (18.7)	13 280 (21.4)	35 595 (23.7)	19 205 (15.3)	1545 (22.6)	16 365 (3.2)	1635 (28.6)	650 (19.2)
≥60	213 935 (78.7)	150 255 (16.3)	4370 (9.4)	4590 (48.9)	18 280 (29.4)	87 015 (57.6)	33 320 (26.6)	335 (4.9)	10 (<0.1)	1300 (22.7)	1040 (30.8)
Income quartile											
<1 (lowest)	131 570 (48.4)	180 505 (19.6)	8430 (18.2)	2275 (24.2)	19 755 (31.8)	31 570 (20.9)	28 050 (22.4)	2650 (38.7)	85 715 (16.7)	1295 (22.6)	770 (22.9)
1 to <2	73 270 (26.9)	214 285 (23.2)	7165 (15.5)	2940 (31.3)	19 550 (31.5)	58 080 (38.5)	29 260 (23.4)	1520 (22.2)	94 110 (18.4)	765 (13.4)	895 (26.5)
2 to <3	31 775 (11.7)	252 440 (27.4)	7415 (16.0)	1515 (16.1)	10 675 (17.2)	31 545 (20.9)	18 285 (14.6)	1155 (16.9)	180 360 (35.2)	895 (15.7)	595 (17.7)
≥3 (highest)	22 205 (8.2)	188 025 (20.4)	5200 (11.2)	1105 (11.8)	4810 (7.7)	21 275 (14.1)	13 250 (10.6)	915 (13.4)	140 100 (27.3)	920 (16.1)	445 (13.2)
Child^c^	10 655 (3.9)	68 085 (7.4)	17 295 (37.4)	1440 (15.3)	5400 (8.7)	7460 (4.9)	33 760 (27.0)	355 (5.2)	60 (<0.1)	1710 (29.9)	605 (17.9)
Missing	2425 (0.9)	19 410 (2.1)	770 (1.7)	115 (1.2)	1940 (3.1)	1070 (0.7)	2595 (2.1)	255 (3.7)	12 475 (2.4)	135 (2.3)	60 (1.8)
Marital status											
Married or registered partner	253 155 (93.1)	340 385 (36.9)	9865 (21.3)	2905 (30.9)	14 985 (24.1)	60 670 (40.2)	24 795 (19.8)	1290 (18.8)	221 650 (43.2)	3250 (56.9)	975 (28.9)
Single	16 975 (6.2)	448 215 (48.6)	30 850 (66.7)	3400 (36.2)	29 275 (47.1)	31 200 (20.7)	70 510 (56.3)	4525 (66.1)	274 535 (53.5)	2390 (41.8)	1530 (45.4)
Divorced	1240 (0.5)	58 805 (6.4)	2920 (6.3)	1070 (11.4)	8445 (13.6)	20 105 (13.3)	10 640 (8.5)	865 (12.6)	14 345 (2.8)	45 (0.8)	370 (10.9)
Widowed	180 (0.1)	69 750 (7.6)	1730 (3.7)	1975 (21.1)	9180 (14.8)	38 495 (25.5)	17 395 (13.9)	125 (1.8)	385 (0.1)	<10 (0.1)	455 (13.5)
Unknown	345 (0.1)	5595 (0.6)	910 (2.0)	35 (0.4)	245 (0.4)	525 (0.3)	1865 (1.5)	40 (0.6)	1910 (0.4)	25 (0.4)	45 (1.3)

Abbreviation: TBI, traumatic brain injury.

^a^ Cells with a sample size of 5 or less and cells in which a value presented could result in the calculation of a sample size of less than 5 in a complementary cell are presented as less than 10.

^b^ Indicates multiple traumas on the day of the first-recorded trauma.

^c^ Persons under the age of 14 years with no income data.

#### Incidence of Posttraumatic Psychopathology

Five-year incidence of psychiatric disorders within each *ICD-10* category is presented in Table 3. Overall, 65 080 males (13.7%) and 92 030 females (10.0%) exhibited some posttraumatic psychopathology. The most common diagnoses for males were substance use (35 160 of 475 280 males [7.4%]), organic (13 580 of 475 280 males [2.9%]), and depressive disorders (12 820 of 475 280 males [2.7%]), and for females, depressive (29 255 of 922 750 females [3.2%]), stress-related (23 305 of 922 750 females [2.5%]), and substance use disorders (22 045 of 922 750 females [2.4%]).

**Table 3.** Incidence of Psychiatric Disorders Within 5 Years of First-Recorded Traumatic Event in Males and Females

Disorders^a^	Individuals, No. (%)									
	Total	Fire or explosion	Transportation accident	Exposure to toxic substance	Medical complications or injury	TBI	Physical assault	Pregnancy-related trauma	Suicide of family member	Multiple traumatic events^b^
**Males**										
Total No.	475 280	67 715	14 360	46 750	145 320	171 200	16 770	3685	3875	5600
None	410 200 (86.3)	64 120 (94.7)	12 860 (89.6)	28 830 (61.7)	129 395 (89.0)	148 200 (86.6)	14 900 (88.8)	3555 (96.4)	3600 (93.0)	4735 (84.6)
Organic	13 580 (2.9)	350 (0.5)	335 (2.3)	2135 (4.6)	5020 (3.5)	5395 (3.2)	90 (0.5)	<10 (0.2)^c^	30 (0.8)	215 (3.9)
Substance use	35 160 (7.4)	2020 (3.0)	730 (5.1)	10 085 (21.6)	6625 (4.6)	13 940 (8.1)	1170 (7.0)	55 (1.5)	80 (2.1)	455 (8.1)
Schizophrenia or psychotic	7455 (1.6)	505 (0.7)	115 (0.8)	3095 (6.6)	1160 (0.8)	2020 (1.2)	410 (2.4)	15 (0.4)	15 (0.4)	125 (2.2)
Manic episode or bipolar	3260 (0.7)	150 (0.2)	60 (0.4)	1420 (3.0)	610 (0.4)	880 (0.5)	80 (0.5)	<10 (0.2)^c^	10 (0.3)	40 (0.7)
Depressive	12 820 (2.7)	575 (0.8)	280 (2.0)	4740 (10.1)	3355 (2.3)	3345 (2.0)	295 (1.8)	25 (0.7)	60 (1.5)	135 (2.4)
Neurotic or somatoform	4970 (1.0)	330 (0.5)	140 (1.0)	1425 (3.1)	1345 (0.9)	1435 (0.8)	210 (1.3)	10 (0.3)	15 (0.4)	55 (1.0)
Stress	11 200 (2.4)	705 (1.0)	275 (1.9)	4910 (10.5)	1600 (1.1)	2960 (1.7)	435 (2.6)	40 (1.1)	115 (3.0)	155 (2.7)
Physiological^d^	945 (0.2)	80 (0.1)	20 (0.1)	195 (0.4)	250 (0.2)	325 (0.2)	45 (0.3)	<10 (0.2)^c^	10 (0.2)	20 (0.4)
Adult personality	6140 (1.3)	410 (0.6)	110 (0.8)	2790 (6.0)	680 (0.5)	1835(1.1)	210 (1.3)	20 (0.6)	15 (0.4)	70 (1.2)
**Females**										
Total No.	922 750	46 280	9385	62 125	150 995	125 200	6845	512 820	5715	3375^a^
None	830 720 (90.0)	43 245 (93.4)	8170 (87.0)	33 725 (54.3)	132 350 (87.7)	108 705 (86.8)	5535 (80.8)	490 985 (95.7)	5210 (91.1)	2800 (83.0)
Organic	16 650 (1.8)	420,(0.9)	505 (5.4)	3075 (5.0)	6850 (4.5)	5350 (4.3)	55 (0.8)	180 (0)	75 (1.3)	140 (4.1)
Substance use	22 045 (2.4)	785 (1.7)	235 (2.5)	8625 (13.9)	3920 (2.6)	4815 (3.8)	580 (8.5)	2815 (0.5)	95 (1.6)	180 (5.3)
Schizophrenia or psychotic	8455 (0.9)	390 (0.8)	65 (0.7)	4085 (6.6)	1240 (0.8)	1190 (0.9)	190 (2.8)	1220 (0.2)	25 (0.5)	50 (1.5)
Manic episode or bipolar	6305 (0.7)	165 (0.4)	55 (0.6)	2975 (4.8)	1130 (0.7)	880 (0.7)	60 (0.9)	985 (0.2)	20 (0.4)	35 (1.0)
Depressive	29 255 (3.2)	760 (1.6)	380 (4.0)	10 400 (16.7)	5890 (3.9)	4070 (3.3)	280 (4.1)	7175 (1.4)	125 (2.2)	175 (5.2)
Neurotic or somatoform	13 785 (1.5)	505 (1.1)	150 (1.6)	3360 (5.4)	2180 (1.4)	1945 (1.6)	200 (2.9)	5325 (1.0)	55 (0.9)	70 (2.1)
Stress	23 305 (2.5)	720 (1.6)	180 (1.9)	10 085 (16.2)	2120 (1.4)	2830 (2.3)	425 (6.2)	6575 (1.3)	235 (4.1)	135 (4.0)
Physiological^d^	5165 (0.6)	240 (0.5)	35 (0.4)	1480 (2.4)	470 (0.3)	805 (0.6)	80 (1.2)	2010 (0.4)	30 (0.5)	20 (0.6)
Adult personality	15 095 (1.6)	475 (1.0)	75 (0.8)	7210 (11.6)	1120 (0.7)	2015 (1.6)	305 (4.5)	3770 (0.7)	55 (1.0)	70 (2.1)

Abbreviation: TBI, traumatic brain injury.

^a^ Disorder classes are not mutually exclusive.

^b^ Indicates multiple traumas on the day of the first-recorded trauma.

^c^ Cells with a sample size of 5 or less and cells in which a value presented could result in the calculation of a sample size of less than 5 in a complementary cell are presented as less than 10.

^d^ Behavioral syndromes associated with physiological disturbances and physical factors (eg, eating disorders).

The PTE associated with the highest incidence of subsequent psychopathology for males (17 920 of 46 750 [38.3%]) and females (28 400 of 62 125 [45.7%]) was exposure to toxic substance. Sex differences were observed in psychopathology after this PTE; substance use disorders were most common among males (10 085 of 46 750 [21.6%]) and depressive (10 400 of 62 125 females [16.7%]) or stress-related (10 085 of 62 125 [16.2%]) disorders were most common among females. Furthermore, females had nearly double the incidence of any psychopathology following physical assault (1310 of 6845 [19.2%]) than males (1870 of 16 770 [11.2%]). Despite sex differences in pregnancy-related trauma, males and females displayed comparable incidence of psychopathology (130 of 3685 males [3.6%]; 21 835 of 512 820 females [4.3%]).

#### Associations Between PTEs and Psychopathology

**Figure 1.**
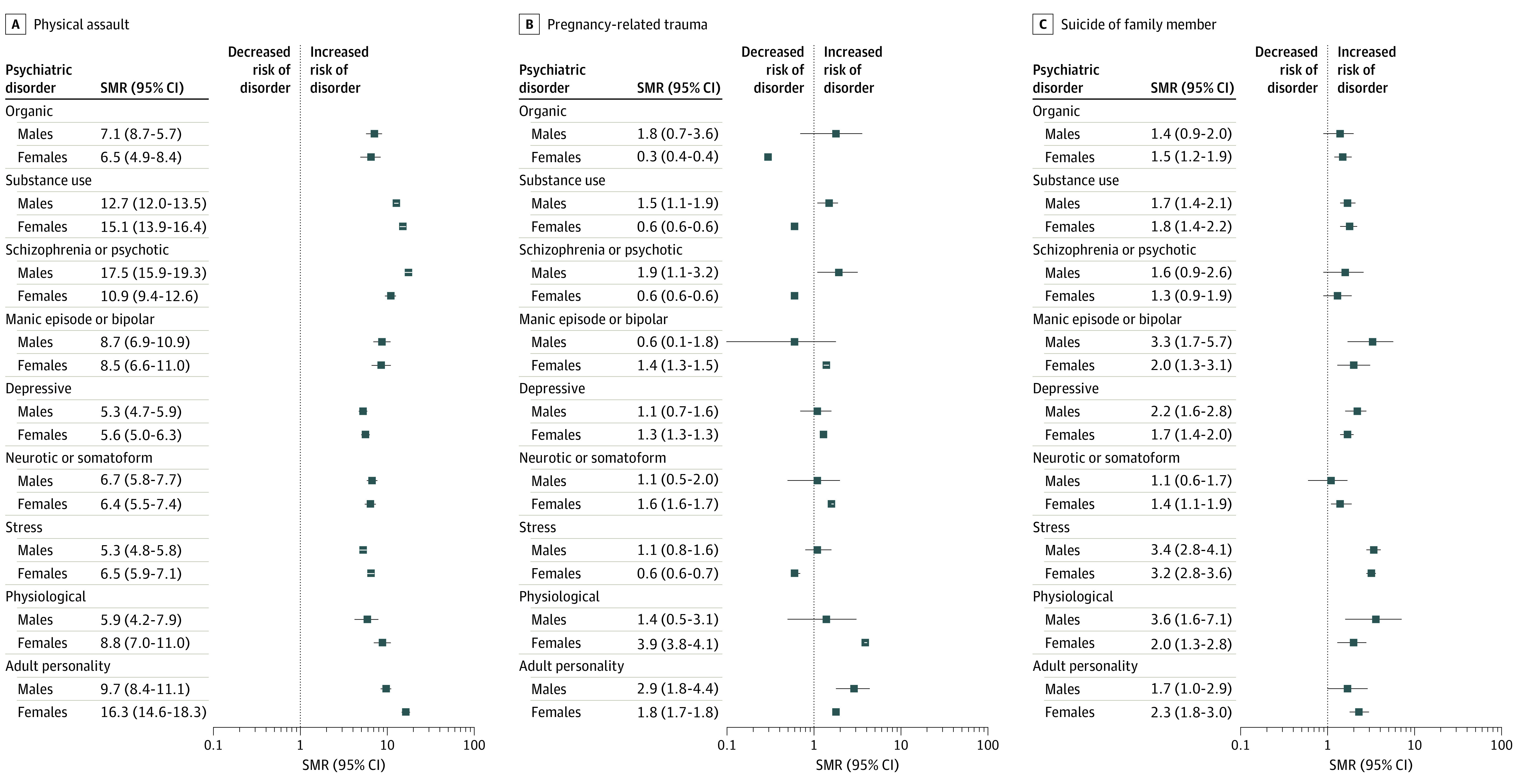
Standardized Morbidity Ratio (SMRs) for Psychiatric Disorder Classes Within 5 Years of First-Recorded Interpersonal Traumatic Events in Full Trauma Cohort Disorder classes are not mutually exclusive. Physiological refers to behavioral syndromes associated with physiological disturbances and physical factors (eg, eating disorders, sleep disorders).

**Figure 2.**
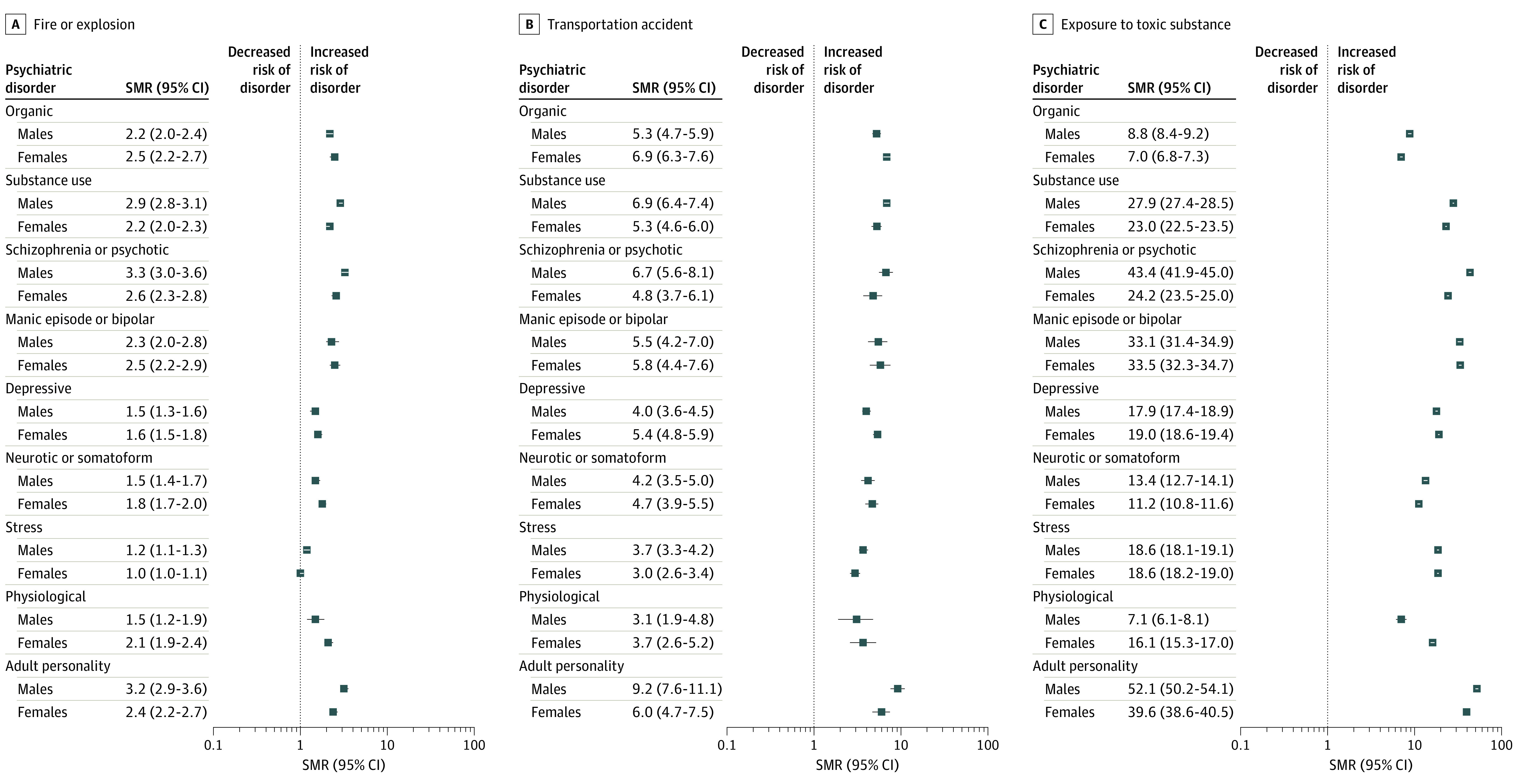
Standardized Morbidity Ratio (SMRs) for Psychiatric Disorder Classes Within 5 Years of First-Recorded Noninterpersonal Traumatic Events in Full Trauma Cohort Disorder classes are not mutually exclusive. Physiological refers to behavioral syndromes associated with physiological disturbances and physical factors (eg, eating disorders, sleep disorders).

Sex-stratified SMRs for associations between PTEs and psychiatric disorder categories are presented in Figure 1 and Figure 2. For individuals experiencing interpersonal PTEs, physical assault had the strongest associations with psychopathology vs a nontraumatic stressor. After physical assault, males and females showed strong associations for elevated rates of substance use disorders (males: SMR, 12.7; 95% CI, 12.0-13.5; females: SMR, 15.1; 95% CI, 13.9-16.4); particularly robust associations also were seen with schizophrenia or psychotic disorders for males (SMR, 17.5; 95% CI, 15.9-19.3) and adult personality disorders for females (SMR, 16.3; 95% CI, 14.6-18.3).

Compared with a nontraumatic stressor, some inverse associations were seen for pregnancy-related trauma in females, specifically for organic, substance use, schizophrenia or psychotic, and stress-related disorders. However, a positive association with physiological disorders was observed. In contrast, for males with pregnancy-related trauma, slightly elevated rates or near-null associations were observed for most psychopathology.

Among noninterpersonal PTEs, exposure to toxic substance had the strongest associations with psychopathology vs a nontraumatic stressor for males and females; both showed the strongest association with adult personality disorders (eg, males: SMR, 52.1; 95% CI, 50.2-54.1). Across sex, large effects indicating elevated rates of psychopathology were also found for multiple PTEs; small to moderate effects were seen for other noninterpersonal PTEs. Across the noninterpersonal PTEs, some consistent sex differences emerged, with males exhibiting larger SMRs for substance use, schizophrenia/psychotic, and adult personality disorders (eg, schizophrenia or psychotic disorder among males exposed to toxic substance: SMR, 43.4; 95% CI, 41.9-45.0) and females exhibiting larger SMRs for depressive disorders (eg, females exposed to toxic substance: SMR, 19.0; 95% CI, 18.6-19.4).

### Results Stratified by Pretrauma Psychopathology

Demographics by PTE types are presented in eTables 2 and 3 in Supplement 1 for males (58 510 of 144 920 individuals [40.4%]) and females (86 410 of 144 920 individuals [59.6%]) with pretrauma psychopathology, and in eTables 4 and 5 in Supplement 1 for males (416 770 of 1 253 110 individuals [33.3%]) and females (836 340 of 1 253 110 individuals [66.7%]) without pretrauma psychopathology. Overall, demographics in these groups mirrored those of the full cohort, except individuals with pretrauma psychopathology more frequently had lower income.

When stratifying by pretrauma psychopathology, the most commonly experienced PTEs for males and females, and patterns of sex differences, were generally consistent with the full cohort. However, for individuals with pretrauma psychopathology, the proportions of males and females experiencing exposure to toxic substance (males, 23.1%; females, 21.5%) and medical complications or injury (males, 28.3%; females, 21.4%) were more comparable. Additionally, for males and females, physical assault was more prevalent for those with (2495 of 58 510 males [4.3%]; 1455 of 86 410 females [1.7%]) vs without pretrauma psychopathology (14 275 of 416 770 males [3.4%]; 5390 of 836 340 females [0.01%]). Although pregnancy-related trauma was the most common PTE for females, it was experienced by fewer females with pretrauma psychopathology (34.5%) vs without pretrauma psychopathology (57.8%).

#### Incidence of Posttraumatic Psychopathology

Five-year incidence of psychiatric diagnoses within each *ICD-10* category are presented in eTables 6 and 7 in Supplement 1 for individuals with and without pretrauma psychopathology. Among individuals without pretrauma psychopathology, incidence rates mirrored the full cohort: 9.0% of males and 6.8% of females developed posttraumatic psychopathology. Aside from substance use disorders, which were more common in males (4.2%) than females (1.3%), incidence was similar across sex. In contrast, among those with pretrauma psychopathology, higher proportions developed psychopathology (males, 53.0%; females, 59.5%), and sex differences were more pronounced. The most common diagnoses for males were substance use (30.1%), depressive (9.3%), and schizophrenia or psychotic (8.7%) disorders and for females, depressive (13.7%), substance use, (12.5%), stress-related (8.7%), and adult personality (9.1%) disorders.

Consistent with the full cohort, the PTE associated with the highest incidence of psychopathology was exposure to toxic substance, particularly among those with pretrauma psychopathology (males, 70.5%; females, 73.6%). Males in this group most commonly developed substance use disorders (45.6%), while females exhibited more variability: depressive (28.0%), substance use (26.8%), adult personality (23.1%), and stress-related (21.0%) disorders. Although incident psychopathology was lower for males and females without pretrauma psychopathology, this pattern of results across sex was similar. Also mirroring the full cohort, even though more males experienced physical assault, females were more likely to develop psychiatric disorders, regardless of pretrauma psychopathology.

For pregnancy-related trauma, differences emerged among females when considering pretrauma psychopathology. Females with pretrauma psychopathology had higher incidence, than those without pretrauma psychopathology, and most commonly exhibited depressive, neurotic or somatoform, stress-related, and adult personality disorders. As stratification resulted in small subsamples (*n* < 10) for males after pregnancy-related trauma, results were not interpreted.

#### PTEs and Psychopathology

Sex-stratified SMRs are visualized in eFigures 1 and 2 in Supplement 1 for individuals with and without pretrauma psychopathology. As in the full cohort, physical assault yielded the largest SMRs among interpersonal PTEs, particularly for those without pretrauma psychopathology. Further, SMRs following physical assault were often larger for females than males in this group, particularly for substance use and adult personality disorders. In individuals with pretrauma psychopathology, SMRs were elevated but generally comparable across sex except for schizophrenia or psychotic disorders (elevations were stronger for males).

SMRs for pregnancy-related PTEs differentiated females with and without pretrauma psychopathology (men’s SMRs were not compared given small subsamples). For example, females with vs without pretrauma psychopathology showed more robust associations with incident depressive, neurotic or somatoform, physiological, stress-related, and adult personality disorders. In contrast, the SMR for stress-related disorders for females without pretrauma psychopathology mirrored the inverse association seen for females overall.

Among noninterpersonal PTEs, exposure to toxic substance had the most robust associations with psychopathology, as in the full cohort. SMRs for this PTE were larger across nearly all psychiatric disorders for individuals without vs with pretrauma psychopathology. Sex-specific differences were also evident across most disorders, although adult personality disorders had the largest SMRs for males and females, regardless of pretrauma psychopathology.

Across most noninterpersonal PTEs, males with pretrauma psychopathology had larger SMRs for organic, substance use, and schizophrenia or psychotic disorders than females, whereas females with pretrauma psychopathology generally had larger SMRs for depressive disorders, mirroring the full cohort. Sex differences were less pronounced among individuals without pretrauma psychopathology, although males frequently had larger SMRs for substance use disorders and females had larger SMRs for depressive disorders, as for those with pretrauma psychopathology.

## Discussion

Psychopathology can take many forms after trauma, but most research has focused on PTSD. To our knowledge, this study is the first to comprehensively investigate sex-related differences in various psychopathology following PTEs using Danish registry data, a unique administrative database resource for trauma epidemiology research. Three main findings emerged. First, consistent with existing research,^23^ males had greater exposure to numerous PTEs than females. Second, various forms of psychopathology onset within 5 years of trauma for males and females, with some patterning by sex. Consistent with general population research,^10^ males most often developed substance use disorders after PTEs, whereas females most frequently exhibited depressive disorders. Third, rates of psychiatric disorders after PTEs (compared with a nontraumatic stressor) were often similar for males and females in the full cohort; however, some sex differences emerged, particularly when considering pretrauma psychopathology. Although PTEs were consistently associated with psychopathology onset regardless of psychiatric history, there was a more nuanced patterning of PTEs and incident psychopathology by sex when stratifying by pretrauma psychopathology.

Although males experienced various PTEs more than females, specific PTEs resulted in psychopathology onset in females that was comparable with or even exceeded that in males. For example, among interpersonal PTEs, incident psychopathology was higher among females following physical assault despite less exposure. Although males have higher rates of assault by strangers, extensive research shows that violence against females is largely perpetrated by individuals known to the victim (eg, intimate partners), which may affect females’ mental health more prominently.^24,25^ Furthermore, although elevated across sex, associations between physical assault and incident psychopathology were particularly elevated for those without pretrauma psychopathology and at times stronger for females than males when compared with a nontraumatic stressor. Among those with pretrauma psychopathology, results for males and females were more congruent, as in the full cohort. These findings underscore the complex and diverse nature of trauma responses and suggest that posttraumatic psychopathology is not wholly a function of preexisting psychopathology, even after severe PTEs like physical assault.^22,26,27^

Although predominantly experienced by females, pregnancy-related trauma had associations with incident psychopathology for males and females. Females with pretrauma psychopathology seemed particularly impacted by this PTE, showing stronger associations across various disorders compared with females without pretrauma psychopathology. Moreover, whereas an inverse association with stress-related disorders emerged overall and in females without pretrauma psychopathology, there was an increased incidence of stress-related disorders among females with pretrauma psychopathology. The perinatal period, marked by physiological and psychosocial changes, is a stress-sensitive juncture that can exacerbate preexisting conditions and introduce new symptoms.^28,29^ This finding holds clinical significance, particularly for the sizeable proportion of females with prior psychopathology who may need care after pregnancy-related PTEs. Considering the comprehensive maternal care in Denmark,^30^ these findings may represent conservative estimates compared with other regions contending with maternal health inequities.^31,32^

A standout noninterpersonal PTE given strong associations with incident psychopathology was exposure to toxic substances, particularly for those without pretrauma psychopathology. Exposure to toxic substances could precipitate certain disorders, particularly those where certain brain areas are affected, and could trigger vulnerabilities.^33,34^ Alternatively, subclinical or undiagnosed psychopathology could precipitate this PTE (eg, self-harm), prompting health care interactions and recording of psychiatric diagnoses. Indeed, various subthreshold psychiatric symptoms are associated with substance misuse—particularly for personality disorders^35^—which aligns with our findings. Risk-taking behavior or self-medication could also explain associations, particularly among those with pretrauma psychopathology.^36^ However, associations between exposure to drugs and medications with abuse potential and psychopathology were less pronounced than for other toxic substances, suggesting these behavioral pathways may be relevant for a smaller portion of the sample (eResults and eTables 8 to 10 in Supplement 1).

### Limitations

This study has limitations. Although we applied qualifiers (eg, hospitalization) to increase the probability that events represented more severe exposures, PTEs were defined by diagnostic codes and might not reflect *Diagnostic and Statistical Manual of Mental Disorders* (Fifth Edition) Criterion A events. We also could not capture all possible PTE experiences and posttraumatic psychopathology, which could introduce certain biases, particularly for high-impact PTEs that disproportionately affect females (eg, sexual violence).^37,38,39^ Underascertainment of PTEs may lead to misclassification; however, results showed that PTEs, as captured in this study, were associated with elevated rates of psychopathology compared with individuals experiencing a nontraumatic stressor (and other possibly uncaptured stressors or PTEs). Psychopathology was also limited to inpatient or outpatient diagnoses, excluding conditions diagnosed by a general practitioner or subthreshold manifestations, possibly underestimating incidence. Indeed, prior research with these registries shows that missing psychiatric diagnoses are likely to be mild.^40^ Furthermore, as almost the entire Danish population makes at least 1 recorded health care contact each year,^41^ it is reasonable to assert that registry data capture a large majority of the population and that severe enough PTEs or psychiatric disorders would likely be discovered during these encounters.

Unaccounted PTEs between first-recorded PTE and the psychopathology window (or beyond) present another limitation. We selected our window for incidence as it balances being reasonably short enough for disorders to be attributed to PTEs and long enough to capture disorders requiring time to develop or be recorded in registries. However, future research should investigate other outcome windows and consider patterns of trauma over time and associations with psychopathology, as a history of trauma is associated with future exposure.^42^ Similarly, although we assessed pretrauma psychopathology, this definition did not encompass psychiatric diagnoses outside the study period. Still, for those with data within the study period and without pretrauma psychopathology, we were able to capture true incidence to a larger degree. More broadly, the generalizability of findings to epidemiological studies based on intentional assessment of lifetime trauma and related psychopathology is limited, and incident psychopathology in this cohort may thus yield lower estimates. Nevertheless, the Danish EHRs provide high-quality, population-based data with an extended temporal scope, enabling more comprehensive examinations of trauma epidemiology.

## Conclusions

Our study demonstrates the wide-ranging psychiatric consequences of trauma in a population-based sample of males and females. Results highlighted some similarities in posttraumatic psychopathology while also uncovering distinct manifestations for males and females. Delving deeper into the sex-specific impacts of trauma could yield more comprehensive etiological models of psychopathology, enhance assessment, and facilitate treatment for individuals who have been exposed to trauma.

## Notes

Supplement 1.
eMethods.

eResults.
**eTable 1.** Trauma Data Sources and Variable Definitions **eTable 2.** Demographic Characteristics of Males With Pretrauma Psychopathology in the Danish Health Registry Trauma Cohort and Comparison Cohort **eTable 3.** Demographic Characteristics of Females With Pretrauma Psychopathology in the Danish Health Registry Trauma Cohort and Comparison Cohort **eTable 4.** Demographic Characteristics of Males Without Pretrauma Psychopathology in the Danish Health Registry Trauma Cohort and Comparison Cohort **eTable 5.** Demographic Characteristics of Females Without Pretrauma Psychopathology in the Danish Health Registry Trauma Cohort and Comparison Cohort **eTable 6.** Incidence of Psychiatric Disorders Within 5 Years of First-Recorded Traumatic Event in Males and Females With Pretrauma Psychopathology **eTable 7.** Incidence of Psychiatric Disorders Within 5 Years of First-Recorded Traumatic Event in Males and Females Without Pretrauma Psychopathology **eFigure 1.** SMRs for Psychiatric Disorder Classes Within 5 Years of First-Recorded Traumatic Event in Individuals With Pretrauma Psychopathology **eFigure 2.** SMRs for Psychiatric Disorder Classes Within 5 Years of First-Recorded Traumatic Event in Individuals Without Pretrauma Psychopathology **eTable 8.** Demographic Characteristics of Males and Females in the Danish Health Registry Trauma Cohort With Exposure to Toxic Substance **eTable 9.** Incidence of Psychiatric Disorders Within 5 Years of First-Recorded Exposure to Toxic Substance in Males and Females **eTable 10.** Standardized Morbidity Ratio (SMR) for Psychiatric Disorder Classes Within 5 Years of First-Recorded Exposure to Toxic Substance in Males and Females
eReferences.

Supplement 2. Data Sharing Statement
