# Supplementary material for: Sex Differences in Psychopathology Following Potentially Traumatic Experiences
Source: JAMA Netw Open. 2024 Feb 22;7(2):e240201. doi: 10.1001/jamanetworkopen.2024.0201 (PMC10884878; doi:10.1001/jamanetworkopen.2024.0201)
Supplement: Supplement 1. — eMethods. eResults. eTable 1. Trauma Data Sources and Variable Definitions eTable 2. Demographic Characteristics of Males With Pretrauma Psychopathology in the Danish Health Registry Trauma Cohort and Comparison Cohort eTable 3. Demographic Characteristics of Females With Pretrauma Psychopathology in the Danish Health Registry Trauma Cohort and Comparison Cohort eTable 4. Demographic Characteristics of Males Without Pretrauma Psychopathology in the Danish Health Registry Trauma Cohort and Comparison Cohort eTable 5. Demographic Characteristics of Females Without Pretrauma Psychopathology in the Danish Health Registry Trauma Cohort and Comparison Cohort eTable 6. Incidence of Psychiatric Disorders Within 5 Years of First-Recorded Traumatic Event in Males and Females With Pretrauma Psychopathology eTable 7. Incidence of Psychiatric Disorders Within 5 Years of First-Recorded Traumatic Event in Males and Females Without Pretrauma Psychopathology eFigure 1. SMRs for Psychiatric Disorder Classes Within 5 Years of First-Recorded Traumatic Event in Individuals With Pretrauma Psychopathology eFigure 2. SMRs for Psychiatric Disorder Classes Within 5 Years of First-Recorded Traumatic Event in Individuals Without Pretrauma Psychopathology eTable 8. Demographic Characteristics of Males and Females in the Danish Health Registry Trauma Cohort With Exposure to Toxic Substance eTable 9. Incidence of Psychiatric Disorders Within 5 Years of First-Recorded Exposure to Toxic Substance in Males and Females eTable 10. Standardized Morbidity Ratio (SMR) for Psychiatric Disorder Classes Within 5 Years of First-Recorded Exposure to Toxic Substance in Males and Females eReferences. [file jamanetwopen-e240201-s001.pdf]

Supplemental Online Content

Kofman YB, Selbe S, Szentkúti P, et al. Sex differences in psychopathology following potentially traumatic experiences. *JAMA Netw Open*. 2024;7(2):e240201.
doi:10.1001/jamanetworkopen.2024.0201

eMethods
eResults
eTable 1. Trauma Data Sources and Variable Definitions
eTable 2. Demographic Characteristics of Men With Pretrauma Psychopathology in the Danish Health Registry Trauma Cohort and Comparison Cohort
eTable 3. Demographic Characteristics of Women With Pretrauma Psychopathology in the Danish Health Registry Trauma Cohort and Comparison Cohort
eTable 4. Demographic Characteristics of Men Without Pretrauma Psychopathology in the Danish Health Registry Trauma Cohort and Comparison Cohort
eTable 5. Demographic Characteristics of Women Without Pretrauma Psychopathology in the Danish Health Registry Trauma Cohort and Comparison Cohort
eTable 6. Incidence of Psychiatric Disorders Within 5 Years of First-Recorded Traumatic Event in Men and Women With Pretrauma Psychopathology
eTable 7. Incidence of Psychiatric Disorders Within 5 Years of First-Recorded Traumatic Event in Men and Women Without Pretrauma Psychopathology
eFigure 1. SMRs for Psychiatric Disorder Classes Within 5 Years of First-Recorded Traumatic Event in Individuals With Pretrauma Psychopathology
eFigure 2. SMRs for Psychiatric Disorder Classes Within 5 Years of First-Recorded Traumatic Event in Individuals Without Pretrauma Psychopathology
eTable 8. Demographic Characteristics of Men and Women in the Danish Health Registry Trauma Cohort With Exposure to Toxic Substance
eTable 9. Incidence of Psychiatric Disorders Within 5 Years of First-Recorded Exposure to Toxic Substance in Men and Women
eTable 10. Standardized Morbidity Ratio (SMR) for Psychiatric Disorder Classes Within 5 Years of First-Recorded Exposure to Toxic Substance in Men and Women
eReferences

This supplemental material has been provided by the authors to give readers additional information about their work.

© 2024 Kofman YB et al. *JAMA Network Open.*

eMethods

Data Sources

Demographic data (age, sex, marital status) were obtained from the Danish Civil Registration System, created in 1968 and updated daily since 1989.[1,2] The Income Statistics Register provided income data.[3]

Psychiatric diagnoses were obtained from the Danish Psychiatric Central Research Register (DPCRR) and supplemented with psychiatric diagnoses recorded only in the Danish National Patient Registry (DNPR) covering all Danish hospitals. The diagnostic system used in these registries starting in 1994 is the International Classification of Diseases, tenth revision (ICD-10).[4] The DPCRR has recorded inpatient psychiatric care since 1969 and began including outpatient care data in 1995.[5] DPCRR data include admission and discharge dates, and one primary and up to 20 secondary discharge diagnoses per entry.[5] Validity studies have demonstrated that numerous mental health conditions can be ascertained accurately using diagnostic codes in the DPCRR.[5,6]

Non-psychiatric discharge diagnoses were collected from the DNPR. The DNPR has recorded inpatient care since 1977 and outpatient hospital and emergency department care since 1995.[6] DNPR data included treatment dates, one primary and up to 20 secondary discharge diagnoses per entry, surgery codes, and procedure codes.[5,7] Multiple studies have documented high validity of discharge diagnostic data for key diagnoses in the registry.[5,7]

Pregnancy trauma data were obtained from the Danish Medical Birth Register (MBR). The MBR has been recording data on all live births and stillbirths in Denmark since 1973.[8] Multiple studies have found reasonable validity, compared to medical record review, for second-trimester miscarriages, and spontaneous deliveries and preeclampsia.[9,10] Moderate validity has also been found for uterine rupture.[11]

Death data were gathered from the Cause of Death Registry, which records cause of death (e.g., natural, suicide), autopsy results, and place and date of death.[12] Both underlying and immediate cause of death were used to define our cohort. A validation study confirmed correct classification of 90% of suicide deaths recorded in this registry.[13]

Additional information on Demographics

Demographic characteristics of participants were recorded for age categories (<16, 16-39, 40-59, and >60 years), income quartiles, with children below age 14 years and those with missing data analyzed in separate categories, and marital status (married/registered partner, single, divorced, widowed, unknown).

Additional information on PTE Categories

Exposure to toxic substance and medical complications/injury have been previously analyzed as a single combined PTE category.[14] In the current study, they were analyzed as two separate PTEs, given the distinct nature of the PTE experiences. If individuals experienced ≥1 event on the same day, they were coded in a multiple PTEs category. Because nearly all such event combinations related to non-interpersonal events, we categorized multiple traumatic events as a non-interpersonal PTE for analysis. The most common event combination was transportation accident and TBI for men and women (45%).

Statistical Analyses

Information about confidence intervals

For SMRs, we calculated 95% confidence intervals (CIs) assuming observed numbers of psychiatric diagnoses followed a Poisson distribution. Exact CIs were calculated if <10 observed psychiatric diagnoses were recorded; otherwise, CIs were based on Byar's approximation of the exact Poisson distribution.[15,16]

Subset analyses for exposure to toxic substance

Secondary subset analyses were conducted for the exposure to toxic substance PTE. In these analyses, we used ICD-10 codes to distinguish between individuals exposed to toxic substances with abuse or overdose potential (e.g., commonly misused drugs and medications; ICD-10: T39-T43, T51) and other toxic substances with less potential for abuse or overdose (e.g., non-medicinal substances and chemicals; ICD-10: T36-T38, T44-T50, T52-T65).

eResults

© 2024 [First author last name and initials or group name] et al. *[Journal name].*

Results for exposure to toxic substances subset analyses

For demographics among different subsets of individuals exposed to toxic substance, see eTable 9. Incidence proportions for exposure to toxic substance, partitioning out toxic exposure to drugs and medications with potential for abuse from other toxic substances, are shown in eTable 10. In the overall cohort, incidence of psychopathology was higher after exposure to a toxic substance with less abuse or overdose potential (men 53%, women 55%) compared to exposure to drugs and medications with abuse or overdose potential (men 23%, women 30%). This pattern was generally reflected in those without pre-trauma psychopathology. Among those with pre-trauma psychopathology, incidence proportion for psychopathology was more comparable between exposure to drugs and medications (men 64%, women 66%) and other toxic substances (66% men, 76% women), although more individuals with pre-trauma psychopathology (vs. those without) subsequently developed psychiatric disorders overall. Regardless of type of toxic substance exposure, for men, the highest incidence proportion was for substance use disorders, particularly for those with pre-trauma psychopathology (41%-47%), while women had varied incidence across psychiatric categories.

SMRs for exposure to toxic substance, partitioning out toxic exposure to drugs and medications with potential for abuse from other toxic substances, are shown in eTable 11. Within the full trauma cohort, SMRs for exposure to other toxic substances were substantially larger in magnitude across all psychiatric categories (men's SMRs from 9.2 [95% CIs=7.7, 11.0] to 80.3 [95% CIs=76.9, 83.9]; women's SMRs from 8.7 [95% CIs=8.3, 9.1] to 46.8 [95% CIs=45.6, 48.0]) compared to SMRs for exposure to drugs or medications with potential for abuse (men's SMRs from 5.0 [95% CIs=3.9, 6.3] to 25.3 [95% CIs=23.4, 27.2]; women's SMRs from 5.6 [95% CIs=5.3, 5.9] to 24.9 [95% CIs=23.6, 26.2]). This pattern persisted regardless of whether individuals had pre-trauma psychopathology; however, discrepancies in effect size between these partitioned PTEs were more pronounced within cohorts of men and women without pre-trauma psychopathology. Associations between exposure to toxic substances and adult personality disorders remained robust for men and women, with the highest SMRs regardless of whether this PTE was split by type of toxic substance or when stratified by pre-trauma psychopathology.

Sex differences emerged for toxic exposure to drugs and medication among those with pre-trauma psychopathology, with men having higher SMRs across most psychiatric disorder categories compared to women. This pattern was reversed for those without pre-trauma psychopathology, with women having higher SMRs for the same PTE across all psychiatric disorder categories compared to men without pre-trauma psychopathology. Within-sex differences were also seen for toxic exposure to drugs and medication. For example, men with pre-trauma psychopathology had higher SMRs for this PTE across most psychiatric categories compared to men without pre-existing psychopathology. Conversely, women with pre-trauma psychopathology had lower SMRs for this PTE across most psychiatric disorder categories compared to women without pre-trauma psychopathology. For those with exposure to other toxic substances (i.e., toxic substances with less potential for abuse), men without pre-trauma psychopathology had higher SMRs across all psychiatric disorders compared to women without pre-trauma psychopathology, except for physiological disorders.

© 2024 [First author last name and initials or group name] et al. *[Journal name].*

eTable 1. Trauma Data Sources and Variable Definitions

Trauma	Source of Data	Variable Codes	Additional Qualifier
Fire or explosion	DNPR; DPCRR	ICD-10 codes for exposure to fire: X00-X08<br>ICD-10 codes for burn treatment: T20-T25, T31	Inpatient, outpatient, ER
Transportation accident[†]	DNPR	EUP2-EUP9, EUM2-EUM9: Mode of transport: bicycle, moped, motorcycle, motor scooter, passenger car, van, pickup truck, lorry, truck, bus, other specified and unspecified<br>EUHE01-EUHE09: Falls, all other than falls on the same level<br>EUHG02, EUHB, EUHC, EUHD: Other accidents: Contact or collision with animal; collapse, breakage and deformation of material; malfunction and loss of control of machinery, equipment and materials; malfunction and loss of control of transport vehicle machinery	Inpatient only with ≥ 2 days hospitalized
Exposure to toxic substance	DNPR; DPCRR	ICD-10 codes for poisoning: T36–T65 and T96–T97	Inpatient only with ≥ 2 days hospitalized
Medical complications/injury	DNPR; DPCRR	ICD-10 codes T15–T35 (except: T20-T25, T31), T66–T95, and T98	Inpatient only with ≥ 2 days hospitalized
Traumatic brain injury	DNPR; DPCRR	ICD-10 codes for intracranial injury: S06	Inpatient and outpatient; ER
Physical assault	DNPR; DPCRR	ICD-10 codes for assault: X92, Y01-Y04	Inpatient and outpatient; ER
Assault with a weapon	DNPR; DPCRR	ICD-10 codes for assault: X93-Y00	Inpatient and outpatient; ER
Pregnancy-related trauma	DMBR	ICD-10 codes: O60-O75	Stillbirth or ≥ 3 days hospitalized following delivery complications
Suicidal death of a family member	DCRS; Cause of Death Registry	ICD-10: X60-X84	
Multiple traumatic events	All available registries	More than one of the above events on the same day	

Abbreviations: DCRS = Danish Civil Registration System; DMBR = Danish Medical Birth Registry; DNPR = Danish National Patients Registry; DPCRR = Danish Psychiatric Central Research Register; ER = emergency room; ICD = International Classification of Diseases.

[†]Accident data available from 2008 onwards only using the Nordic Classification of External Causes of Injury.

© 2024 [First author last name and initials or group name] et al. *[Journal name].*

eTable 2. Demographic Characteristics of Men With Pretrauma Psychopathology in the Danish Health Registry Trauma Cohort and Comparison Cohort

Variable	Total men (comparison cohort; N=7,970)	Total men (trauma cohort; N=58,510)	Fire/ explosion (n=3,980)	Transportation accident (n=1,750)	Exposure to toxic substance (n=13,490)	Medical complications/injury (n=16,560)	TBI (n=19,030)	Physical assault (n=2,495)	Pregnancy-related trauma (n=175)	Suicide of family member (n=240)	Multiple traumatic events[a] (n=795)
	n (%)	n (%)	n (%)	n (%)	n (%)	n (%)	n (%)	n (%)	n (%)	n (%)	n (%)
**Age (years)**											
< 16	945 (11.8)	3,575 (6.1)	590 (14.9)	160 (9.0)	295 (2.2)	545 (3.3)	1,595 (8.4)	225 (9.1)	<10	115 (49.2)	50 (6.0)
16-39	465 (5.8)	17,875 (30.6)	1,795 (45.2)	560 (31.9)	4,935 (36.6)	2,290 (13.8)	6,355 (33.4)	1,465 (58.8)	135 (77.5)	40 (17.2)	300 (37.6)
40-59	800 (10.0)	18,985 (32.5)	1,140 (28.7)	525 (30.1)	5,195 (38.5)	4,895 (29.6)	6,190 (32.5)	690 (27.6)	40 (22.5)	45 (18.1)	270 (33.8)
≥ 60	5,765 (72.3)	18,075 (30.9)	450 (11.3)	505 (29.0)	3,065 (22.7)	8,825 (53.3)	4,895 (25.7)	115 (4.6)	<10	35 (15.5)	180 (22.5)
**Income quartile**											
<Q1(lowest)	2,435 (30.6)	13,925 (23.8)	995 (25.1)	485 (27.7)	3,160 (23.4)	3,225 (19.5)	4,635 (24.4)	1,095 (43.9)	30 (18.5)	65 (28.2)	230 (29.2)
Q1 - < Q2	2,785 (34.9)	23,710 (40.5)	1,225 (30.8)	620 (35.4)	5,800 (43.0)	7,485 (45.2)	7,470 (39.3)	720 (28.8)	55 (32.4)	35 (14.7)	300 (37.7)
Q2 - < Q3	1,100 (13.8)	10,645 (18.2)	700 (17.6)	315 (17.9)	2,660 (19.7)	3,130 (18.9)	3,330 (17.5)	315 (12.6)	45 (26.0)	20 (8.0)	130 (16.6)
≥Q3 (highest)	885 (11.1)	7,080 (12.1)	530 (13.3)	195 (11.1)	1,595 (11.8)	2,210 (13.4)	2,180 (11.5)	225 (9.0)	40 (23.1)	20 (8.4)	85 (10.7)
Child[b]	635 (8.0)	2,570 (4.4)	475 (11.9)	125 (7.0)	140 (1.0)	435 (2.6)	1,190 (6.2)	95 (3.8)	<10	80 (34.5)	35 (4.4)
Missing	125 (1.6)	580 (1.0)	50 (1.3)	15 (0.9)	140 (1.0)	75 (0.4)	225 (1.2)	50 (2.0)	<10	15 (6.3)	10 (1.4)
**Marital status**											
Married/ registered partner	6,555 (82.2)	15,015 (25.7)	715 (18.0)	440 (25.3)	3,215 (23.8)	6,115 (36.9)	3,910 (20.6)	305 (12.3)	70 (39.3)	80 (34.0)	155 (19.6)
Single	1,355 (17.0)	28,840 (49.3)	2,645 (66.5)	945 (54.1)	6,925 (51.3)	5,450 (32.9)	10,300 (54.1)	1,855 (74.2)	90 (53.2)	155 (64.7)	470 (59.4)
Divorced	50 (0.7)	10,935 (18.7)	540 (13.6)	270 (15.4)	2,725 (20.2)	3,290 (19.9)	3,655 (19.2)	305 (12.3)	15 (7.5)	<10	130 (16.2)
Widowed	<10	3,605 (6.2)	65 (1.6)	90 (5.2)	595 (4.4)	1,685 (10.2)	1,115 (5.8)	15 (0.6)	<10	<10	40 (4.8)
Unknown	<10	110 (0.2)	10 (0.3)	<10	25 (0.2)	15 (0.1)	45 (0.2)	15 (0.6)	<10	<10	<10

Notes. Cells with a sample size of five or less and cells in which a value presented could result in the calculation of a sample size of less than 5 in a complementary cell are presented as <10. TBI = traumatic brain injury. Men included in the pregnancy-related trauma category are those who experienced the stillbirth of a child.
[a]Indicates multiple traumas on the day of the first-recorded trauma. [b]Persons under the age of 14 with no income data.

© 2024 [First author last name and initials or group name] et al. *[Journal name].*

eTable 3. Demographic Characteristics of Women With Pretrauma Psychopathology in the Danish Health Registry Trauma Cohort and Comparison Cohort

Variable	Total women (comparison cohort; N=14,875)	Total women (trauma cohort; N=86,410)	Fire/ explosion (n=3,005)	Transportation accident (n=1,180)	Exposure to toxic substance (n=18,605)	Medical complications/i njury (n=18,470)	TBI (n=13,170)	Physical assault (n=1,455)	Pregnancy-related trauma (n=29,780)	Suicide of family member (n=285)	Multiple traumatic events[a] (n=465)
	n (%)	n (%)	n (%)	n (%)	n (%)	n (%)	n (%)	n (%)	n (%)	n (%)	n (%)
**Age (years)**											
< 16	430 (2.9)	3,040 (3.5)	290 (9.6)	45 (4.0)	1,225 (6.6)	385 (2.1)	930 (7.0)	80 (5.6)	10 (0.0)	50 (17.7)	20 (4.8)
16-39	370 (2.5)	44,275 (51.2)	1,300 (43.2)	235 (19.9)	7,005 (37.7)	2,155 (11.7)	3,920 (29.8)	870 (59.7)	28,575 (96.0)	65 (22.6)	155 (33.0)
40-59	1,810 (12.2)	15,920 (18.4)	880 (29.2)	240 (20.5)	5,400 (29.0)	4,635 (25.1)	2,950 (22.4)	425 (29.2)	1,190 (4.0)	85 (29.3)	120 (26.1)
≥ 60	12,270 (82.5)	23,175 (26.8)	540 (18.0)	655 (55.6)	4,975 (26.7)	11,295 (61.2)	5,370 (40.8)	80 (5.5)	<10	85 (30.4)	165 (36.1)
**Income quartile**											
<Q1 (lowest)	8,285 (55.7)	24,570 (28.4)	830 (27.6)	300 (25.5)	6,180 (33.2)	3,815 (20.7)	3,515 (26.7)	570 (39.2)	9,120 (30.6)	100 (35.7)	140 (30.5)
Q1 - < Q2	4,435 (29.8)	31,990 (37.0)	1,030 (34.3)	545 (46.3)	6,945 (37.3)	9,180 (49.7)	5,420 (41.1)	430 (29.7)	8,175 (27.5)	65 (22.3)	200 (43.6)
Q2 - < Q3	1,220 (8.2)	18,780 (21.7)	620 (20.6)	190 (16.3)	3,530 (19.0)	3,610 (19.5)	2,440 (18.5)	240 (16.6)	8,030 (27.0)	55 (19.8)	65 (14.3)
≥Q3 (highest)	570 (3.8)	8,750 (10.1)	260 (8.6)	100 (8.5)	1,310 (7.1)	1,505 (8.1)	1,040 (7.9)	145 (10.1)	4,330 (14.5)	25 (8.1)	35 (7.6)
Child[b]	275 (1.8)	1,530 (1.8)	230 (7.7)	35 (2.9)	280 (1.5)	295 (1.6)	605 (4.6)	30 (2.1)	<10	35 (13.1)	15 (3.2)
Missing	90 (0.6)	785 (0.9)	35 (1.2)	<10	360 (1.9)	65 (0.3)	155 (1.2)	35 (2.3)	130 (0.4)	<10	<10
**Marital status**											
Married/ registered partner	14,025 (94.3)	22,505 (26.0)	625 (20.9)	280 (23.9)	4,225 (22.7)	5,450 (29.5)	2,610 (19.8)	210 (14.4)	8,820 (29.6)	190 (66.8)	95 (20.7)
Single	735 (4.9)	40,670 (47.1)	1,655 (55.1)	345 (29.2)	8,560 (46.0)	3,945 (21.4)	5,355 (40.7)	950 (65.2)	19,590 (65.8)	85 (30.7)	185 (40.2)
Divorced	100 (0.7)	12,210 (14.1)	495 (16.5)	230 (19.7)	3,605 (19.4)	3,905 (21.1)	2,325 (17.6)	265 (18.3)	1,280 (4.3)	<10	100 (21.4)
Widowed	10 (0.1)	10,885 (12.6)	220 (7.3)	320 (27.1)	2,195 (11.8)	5,155 (27.9)	2,865 (21.8)	25 (1.8)	20 (0.1)	<10	80 (17.1)
Unknown	<10	145 (0.2)	10 (0.3)	<10	25 (0.1)	15 (0.1)	15 (0.1)	<10	75 (0.3)	<10	<10

Notes. Cells with a sample size of five or less and cells in which a value presented could result in the calculation of a sample size of less than 5 in a complementary cell are presented as <10. TBI = traumatic brain injury.

[a]Indicates multiple traumas on the day of the first-recorded trauma. [b]Persons under the age of 14 with no income data.

© 2024 [First author last name and initials or group name] et al. *[Journal name].*

eTable 4. Demographic Characteristics of Men Without Pretrauma Psychopathology in the Danish Health Registry Trauma Cohort and Comparison Cohort

Variable	Total men (comparison cohort; N=140,675)	Total men (trauma cohort; N=416,770)	Fire/ explosion (n=63,735)	Transportation accident (n=12,610)	Exposure to toxic substance (n=33,265)	Medical complications/injury (n=128,760)	TBI (n=152,170)	Physical assault (n=14,275)	Pregnancy-related trauma (n=3,510)	Suicide of family member (n=3,635)	Multiple traumatic events[a] (n=4,805)
	n (%)	n (%)	n (%)	n (%)	n (%)	n (%)	n (%)	n (%)	n (%)	n (%)	n (%)
**Age (years)**											
< 16	15,680 (11.1)	97,780 (23.5)	24,750 (38.8)	2,360 (18.7)	6,120 (18.4)	11,345 (8.8)	48,800 (32.1)	1,400 (9.8)	<10	1,995 (54.9)	1,015 (21.1)
16-39	7,115 (5.1)	127,880 (30.7)	23,500 (36.9)	3,425 (27.2)	10,100 (30.4)	22,900 (17.8)	53,055 (34.9)	10,020 (70.2)	2,875 (81.9)	400 (11.0)	1,600 (33.3)
40-59	18,380 (13.1)	82,495 (19.8)	11,805 (18.5)	3,595 (28.5)	7,805 (23.5)	29,760 (23.1)	24,670 (16.2)	2,430 (17.0)	625 (17.8)	645 (17.8)	1,160 (24.2)
≥ 60	99,500 (70.7)	108,610 (26.1)	3,685 (5.8)	3,230 (25.6)	9,240 (27.8)	64,750 (50.3)	25,645 (16.9)	425 (3.0)	10 (0.3)	590 (16.3)	1,030 (21.5)
**Income quartile**											
<Q1 (lowest)	36,155 (25.7)	79,480 (19.1)	9,275 (14.6)	2,720 (21.6)	6,400 (19.2)	22,980 (17.8)	30,205 (19.9)	5,840 (40.9)	315 (9.0)	665 (18.3)	1,080 (22.5)
Q1 - < Q2	39,705 (28.2)	84,255 (20.2)	7,240 (11.4)	2,325 (18.4)	8,500 (25.6)	35,230 (27.4)	26,770 (17.6)	2,565 (18.0)	475 (13.5)	235 (6.5)	915 (19.0)
Q2 - < Q3	21,570 (15.3)	63,785 (15.3)	8,045 (12.6)	2,110 (16.7)	5,630 (16.9)	24,020 (18.7)	20,245 (13.3)	1,960 (13.7)	845 (24.0)	230 (6.3)	705 (14.6)
≥Q3 (highest)	29,525 (21.0)	96,095 (23.1)	15,320 (24.0)	3,270 (25.9)	6,815 (20.5)	35,440 (27.5)	28,795 (18.9)	2,750 (19.3)	1,840 (52.3)	705 (19.3)	1,165 (24.3)
Child[b]	12,195 (8.7)	87,375 (21.0)	22,995 (36.1)	1,975 (15.7)	5,495 (16.5)	10,115 (7.9)	43,555 (28.6)	685 (4.8)	<10	1,705 (46.9)	855 (17.7)
Missing	1,530 (1.1)	5,775 (1.4)	865 (1.4)	210 (1.7)	425 (1.3)	980 (0.8)	2,600 (1.7)	475 (3.3)	40 (1.1)	95 (2.7)	90 (1.8)
**Marital status**											
Married/ registered partner	118,540 (84.3)	139,155 (33.4)	14,810 (23.2)	4,705 (37.3)	11,410 (34.3)	67,290 (52.3)	33,970 (22.3)	2,320 (16.3)	1,900 (54.1)	1,265 (34.8)	1,480 (30.8)
Single	20,950 (14.9)	221,615 (53.2)	44,230 (69.4)	6,295 (49.9)	16,160 (48.6)	37,305 (29.0)	100,390 (66.0)	10,840 (75.9)	1,440 (40.9)	2,305 (63.4)	2,660 (55.3)
Divorced	770 (0.5)	31,640 (7.6)	3,020 (4.7)	1,055 (8.4)	3,415 (10.3)	12,585 (9.8)	10,070 (6.6)	885 (6.2)	165 (4.7)	25 (0.7)	415 (8.7)
Widowed	45 (0.0)	19,595 (4.7)	575 (0.9)	495 (3.9)	2,065 (6.2)	10,740 (8.3)	5,480 (3.6)	55 (0.4)	<10	<10	180 (3.7)
Unknown	370 (0.3)	4,760 (1.1)	1,105 (1.7)	65 (0.5)	210 (0.6)	835 (0.7)	2,260 (1.5)	175 (1.2)	<10	35 (1.0)	70 (1.5)

Notes. Cells with a sample size of five or less and cells in which a value presented could result in the calculation of a sample size of less than 5 in a complementary cell are presented as <10. TBI = traumatic brain injury. Men included in the pregnancy-related trauma category are those who experienced the stillbirth of a child.
[a]Indicates multiple traumas on the day of the first-recorded trauma. [b]Persons under the age of 14 with no income data.

© 2024 [First author last name and initials or group name] et al. *[Journal name].*

eTable 5. Demographic Characteristics of Women Without Pretrauma Psychopathology in the Danish Health Registry Trauma Cohort and Comparison Cohort

Variable	Total women (comparison cohort; N=257,020)	Total women (trauma cohort; N=836,340)	Fire/ explosion (n=43,270)	Transportation accident (n=8,210)	Exposure to toxic substance (n=43,520)	Medical complications/injury (n=132,525)	TBI (n=112,035)	Physical assault (n=5,390)	Pregnancy-related trauma (n=483,040)	Suicide of family member (n=5,435)	Multiple traumatic events[a] (n=2,910)
	n (%)	n (%)	n (%)	n (%)	n (%)	n (%)	n (%)	n (%)	n (%)	n (%)	n (%)
**Age (years)**											
< 16	12,895 (5.0)	78,570 (9.4)	18,245 (42.2)	1,575 (19.2)	8,645 (19.9)	8,355 (6.3)	38,155 (34.1)	685 (12.7)	210 (0.0)	2,000 (36.8)	700 (24.1)
16-39	5,165 (2.0)	548,110 (65.5)	13,780 (31.8)	1,185 (14.4)	13,690 (31.5)	17,315 (13.1)	29,675 (26.5)	3,335 (61.8)	467,650 (96.8)	670 (12.3)	810 (27.9)
40-59	37,295 (14.5)	82,575 (9.9)	7,420 (17.1)	1,510 (18.4)	7,880 (18.1)	31,140 (23.5)	16,255 (14.5)	1,120 (20.8)	15,170 (3.1)	1,550 (28.6)	525 (18.1)
≥ 60	201,665 (78.5)	127,085 (15.2)	3,830 (8.9)	3,935 (48.0)	13,305 (30.6)	75,715 (57.1)	27,950 (24.9)	255 (4.7)	10 (0.0)	1,210 (22.3)	870 (29.9)
**Income quartile**											
<Q1 (lowest)	123,285 (48.0)	155,930 (18.6)	7,600 (17.6)	1,975 (24.0)	13,575 (31.2)	27,750 (20.9)	24,535 (21.9)	2,075 (38.5)	76,600 (15.9)	1,195 (22.0)	630 (21.6)
Q1 - < Q2	68,830 (26.8)	182,295 (21.8)	6,135 (14.2)	2,395 (29.2)	12,605 (29.0)	48,905 (36.9)	23,840 (21.3)	1,085 (20.2)	85,930 (17.8)	700 (12.9)	695 (23.8)
Q2 - < Q3	30,550 (11.9)	233,660 (27.9)	6,800 (15.7)	1,320 (16.1)	7,145 (16.4)	27,935 (21.1)	15,845 (14.1)	915 (16.9)	172,330 (35.7)	840 (15.4)	530 (18.2)
≥Q3 (highest)	21,635 (8.4)	179,275 (21.4)	4,945 (11.4)	1,005 (12.3)	3,495 (8.0)	19,770 (14.9)	12,210 (10.9)	770 (14.2)	135,770 (28.1)	895 (16.5)	410 (14.2)
Child[b]	10,380 (4.0)	66,555 (8.0)	17,060 (39.4)	1,405 (17.1)	5,120 (11.8)	7,165 (5.4)	33,155 (29.6)	325 (6.0)	60 (0.0)	1,675 (30.8)	590 (20.3)
Missing	2,335 (0.9)	18,625 (2.2)	735 (1.7)	105 (1.3)	1,580 (3.6)	1,005 (0.8)	2,445 (2.2)	220 (4.1)	12,350 (2.6)	130 (2.4)	55 (1.9)
**Marital status**											
Married/ registered partner	239,130 (93.0)	317,880 (38.0)	9,240 (21.3)	2,625 (32.0)	10,765 (24.7)	55,220 (41.7)	22,185 (19.8)	1,080 (20.0)	212,830 (44.1)	3,065 (56.4)	880 (30.2)
Single	16,240 (6.3)	407,545 (48.7)	29,195 (67.5)	3,060 (37.3)	20,715 (47.6)	27,255 (20.6)	65,155 (58.2)	3,575 (66.4)	254,945 (52.8)	2,300 (42.4)	1,345 (46.2)
Divorced	1,140 (0.4)	46,595 (5.6)	2,425 (5.6)	840 (10.2)	4,840 (11.1)	16,200 (12.2)	8,320 (7.4)	600 (11.1)	13,065 (2.7)	40 (0.7)	270 (9.3)
Widowed	170 (0.1)	58,865 (7.0)	1,515 (3.5)	1,655 (20.2)	6,980 (16.0)	33,340 (25.2)	14,530 (13.0)	100 (1.9)	365 (0.1)	<10	375 (12.9)
Unknown	340 (0.1)	5,450 (0.7)	900 (2.1)	30 (0.4)	220 (0.5)	515 (0.4)	1,850 (1.6)	35 (0.7)	1,830 (0.4)	25 (0.5)	40 (1.4)

Notes. Cells with a sample size of five or less and cells in which a value presented could result in the calculation of a sample size of less than 5 in a complementary cell are presented as <10. TBI = traumatic brain injury.
[a]Indicates multiple traumas on the day of the first-recorded trauma. [b]Persons under the age of 14 with no income data.

© 2024 [First author last name and initials or group name] et al. *[Journal name].*

eTable 6. Incidence of Psychiatric Disorders Within 5 Years of First-Recorded Traumatic Event in Men and Women With Pretrauma Psychopathology

**Men**	Total men (N=58,510)	Fire/ explosion (n=3,980)	Transportation accident (n=1,750)	Exposure to toxic substance (n=13,490)	Medical complication/ injury (n=16,560)	TBI (n=19,030)	Physical assault (n=2,495)	Pregnancy-related trauma (n=175)	Suicide of family member (n=240)	Multiple traumatic events[a] (n=795)
	n (%)	n (%)	n (%)	n (%)	n (%)	n (%)	n (%)	n (%)	n (%)	n (%)
None	31030 (53.0)	2750 (69.1)	1185 (67.7)	3975 (29.5)	10660 (64.4)	10065 (52.9)	1640 (65.6)	135 (78.6)	185 (76.9)	440 (55.6)
Organic	4810 (8.2)	120 (3.0)	105 (5.9)	1115 (8.3)	1620 (9.8)	1715 (9.0)	55 (2.2)	<10	10 (3.4)	70 (8.9)
Substance Use	17585 (30.1)	765 (19.3)	325 (18.6)	6150 (45.6)	3090 (18.7)	6410 (33.7)	575 (23.0)	20 (10.4)	25 (9.7)	225 (28.1)
Schizophrenia/ psychotic	5080 (8.7)	305 (7.7)	65 (3.8)	2375 (17.6)	810 (4.9)	1175 (6.2)	255 (10.1)	<10	<10	85 (10.6)
Manic episode/bipolar	2080 (3.6)	75 (1.9)	35 (2.1)	1015 (7.5)	360 (2.2)	525 (2.8)	45 (1.8)	<10	<10	25 (3.0)
Depressive	5445 (9.3)	190 (4.8)	105 (6.0)	2465 (18.3)	1130 (6.8)	1345 (7.1)	140 (5.6)	<10	10 (4.2)	50 (6.2)
Neurotic/somatoform	2210 (3.8)	125 (3.2)	55 (3.1)	895 (6.6)	410 (2.5)	595 (3.1)	100 (4.1)	<10	<10	25 (3.0)
Stress	4150 (7.1)	190 (4.8)	90 (5.1)	2095 (15.5)	520 (3.1)	1000 (5.3)	170 (6.8)	10 (6.4)	20 (9.2)	50 (6.0)
Physiological[b]	330 (0.6)	20 (0.6)	<10	115 (0.9)	60 (0.4)	105 (0.5)	20 (0.7)	<10	<10	<10
Adult personality	3330 (5.7)	160 (4.0)	70 (3.9)	1735 (12.9)	335 (2.0)	850 (4.5)	135 (5.3)	10 (5.2)	<10	35 (4.4)

**Women**	Total women (N=86,410)	Fire/ explosion (n=3,005)	Transportation accident (n=1,180)	Exposure to toxic substance (n=18,605)	Medical complication/ injury (n=18,470)	TBI (n=13,170)	Physical assault (n=1,455)	Pregnancy-related trauma (n=29,780)	Suicide of family member (n=285)	Multiple traumatic events[a] (n=465)
	n (%)	n (%)	n (%)	n (%)	n (%)	n (%)	n (%)	n (%)	n (%)	n (%)
None	51435 (59.5)	1925 (64.0)	740 (62.7)	4910 (26.4)	11620 (62.9)	7445 (56.5)	830 (57.0)	23560 (79.1)	175 (61.5)	230 (50.1)
Organic	5690 (6.6)	140 (4.7)	150 (12.7)	1400 (7.5)	2220 (12.0)	1645 (12.5)	25 (1.8)	45 (0.2)	20 (6.4)	45 (9.7)
Substance Use	10765 (12.5)	325 (10.9)	115 (9.7)	4990 (26.8)	1800 (9.7)	2185 (16.6)	295 (20.4)	935 (3.1)	25 (9.2)	95 (20.3)
Schizophrenia/ Psychotic	5485 (6.3)	255 (8.4)	40 (3.4)	3005 (16.2)	795 (4.3)	665 (5.1)	125 (8.7)	565 (1.9)	10 (3.5)	25 (5.8)
Manic episode/bipolar	3890 (4.5)	90 (3.0)	30 (2.5)	2055 (11.0)	690 (3.7)	510 (3.9)	45 (3.1)	435 (1.5)	10 (3.9)	25 (5.6)
Depressive	11820 (13.7)	295 (9.9)	145 (12.2)	5220 (28.0)	2290 (12.4)	1560 (11.8)	130 (9.0)	2080 (7.0)	30 (11.3)	70 (14.7)
Neurotic/somatoform	5510 (6.4)	190 (6.3)	55 (4.8)	1995 (10.7)	820 (4.4)	720 (5.5)	100 (6.8)	1585 (5.3)	15 (5.7)	30 (6.3)
Stress	7495 (8.7)	205 (6.9)	70 (5.9)	3915 (21.0)	690 (3.7)	850 (6.4)	195 (13.3)	1475 (5.0)	45 (16.6)	50 (10.8)
Physiological[b]	2140 (2.5)	90 (3.0)	15 (1.3)	905 (4.9)	175 (0.9)	255 (2.0)	40 (2.8)	645 (2.2)	<10	10 (1.7)
Adult personality	7885 (9.1)	225 (7.5)	40 (3.3)	4290 (23.1)	590 (3.2)	920 (7.0)	170 (11.7)	1600 (5.4)	15 (4.9)	40 (9.1)

Notes. Cells with a sample size of five or less and cells in which a value presented could result in the calculation of a sample size of less than 5 in a complementary cell are presented as <10. Disorder classes are not mutually exclusive. TBI = traumatic brain injury.
[a]Indicates multiple traumas on the day of the first-recorded trauma. [b]Behavioral syndromes associated with physiological disturbances and physical factors (e.g., eating disorders).

© 2024 [First author last name and initials or group name] et al. *[Journal name].*

eTable 7. Incidence of Psychiatric Disorders Within 5 Years of First-Recorded Traumatic Event in Men and Women Without Pretrauma Psychopathology

**Men**	Total men (N=416,770)	Fire/ explosion (n=63,735)	Transportation accident (n=12,610)	Exposure to toxic substance (n=33,265)	Medical complication/ injury (n=128,760)	TBI (n=152,170)	Physical assault (n=14,275)	Pregnancy-related trauma (n=3,510)	Suicide of family member (n=3,635)	Multiple traumatic events[a] (n=4,805)
	n (%)	n (%)	n (%)	n (%)	n (%)	n (%)	n (%)	n (%)	n (%)	n (%)
None	379165 (91.0)	61370 (96.3)	11680 (92.6)	24855 (74.7)	118735 (92.2)	138135 (90.8)	13260 (92.9)	3420 (97.3)	3420 (94.0)	4295 (89.3)
Organic	8770 (2.1)	235 (0.4)	230 (1.8)	1020 (3.1)	3395 (2.6)	3680 (2.4)	35 (0.3)	<10	25 (0.6)	145 (3.0)
Substance Use	17575 (4.2)	1255 (2.0)	400 (3.2)	3935 (11.8)	3535 (2.7)	7530 (4.9)	595 (4.2)	35 (1.1)	60 (1.6)	230 (4.8)
Schizophrenia/ psychotic	2375 (0.6)	200 (0.3)	50 (0.4)	720 (2.2)	345 (0.3)	845 (0.6)	155 (1.1)	10 (0.3)	10 (0.3)	40 (0.8)
Manic episode/bipolar	1180 (0.3)	75 (0.1)	25 (0.2)	410 (1.2)	255 (0.2)	355 (0.2)	35 (0.2)	<10	<10	15 (0.4)
Depressive	7375 (1.8)	380 (0.6)	175 (1.4)	2275 (6.8)	2225 (1.7)	2000 (1.3)	155 (1.1)	20 (0.6)	50 (1.3)	90 (1.8)
Neurotic/somatoform	2755 (0.7)	205 (0.3)	85 (0.7)	530 (1.6)	940 (0.7)	840 (0.6)	110 (0.8)	<10	10 (0.3)	35 (0.7)
Stress	7050 (1.7)	515 (0.8)	185 (1.5)	2815 (8.5)	1080 (0.8)	1960 (1.3)	265 (1.9)	30 (0.9)	95 (2.6)	105 (2.2)
Physiological[b]	615 (0.1)	55 (0.1)	20 (0.2)	80 (0.2)	185 (0.1)	220 (0.1)	25 (0.2)	<10	<10	15 (0.3)
Adult personality	2810 (0.7)	250 (0.4)	45 (0.3)	1055 (3.2)	345 (0.3)	985 (0.6)	80 (0.5)	10 (0.3)	10 (0.3)	35 (0.7)

**Women**	Total women (N=836,340)	Fire/ explosion (n=43,270)	Transportation accident (n=8,210)	Exposure to toxic substance (n=43,520)	Medical complication/ injury (n=132,525)	TBI (n=112,035)	Physical assault (n=5,390)	Pregnancy-related trauma (n=483,040)	Suicide of family member (n=5,435)	Multiple traumatic events[a] (n=2,910)
	n (%)	n (%)	n (%)	n (%)	n (%)	n (%)	n (%)	n (%)	n (%)	n (%)
None	779285 (93.2)	41320 (95.5)	7430 (90.5)	28815 (66.2)	120730 (91.1)	101260 (90.4)	4705 (87.2)	467425 (96.8)	5035 (92.7)	2565 (88.2)
Organic	10955 (1.3)	280 (0.6)	355 (4.3)	1675 (3.8)	4630 (3.5)	3705 (3.3)	30 (0.5)	130 (0.0)	55 (1.0)	95 (3.2)
Substance Use	11280 (1.3)	460 (1.1)	120 (1.5)	3635 (8.4)	2120 (1.6)	2630 (2.3)	285 (5.2)	1880 (0.4)	70 (1.3)	85 (2.9)
Schizophrenia/ Psychotic	2970 (0.4)	140 (0.3)	25 (0.3)	1080 (2.5)	450 (0.3)	520 (0.5)	65 (1.2)	655 (0.1)	15 (0.3)	25 (0.8)
Manic episode/bipolar	2415 (0.3)	75 (0.2)	25 (0.3)	920 (2.1)	440 (0.3)	365 (0.3)	15 (0.3)	550 (0.1)	10 (0.2)	10 (0.3)
Depressive	17435 (2.1)	465 (1.1)	235 (2.9)	5185 (11.9)	3600 (2.7)	2510 (2.2)	150 (2.8)	5090 (1.1)	95 (1.7)	110 (3.7)
Neurotic/somatoform	8275 (1.0)	315 (0.7)	90 (1.1)	1370 (3.1)	1360 (1.0)	1220 (1.1)	100 (1.9)	3740 (0.8)	40 (0.7)	40 (1.4)
Stress	15810 (1.9)	510 (1.2)	110 (1.4)	6175 (14.2)	1430 (1.1)	1980 (1.8)	235 (4.3)	5100 (1.1)	185 (3.4)	85 (3.0)
Physiological[b]	3025 (0.4)	150 (0.3)	20 (0.2)	575 (1.3)	295 (0.2)	545 (0.5)	40 (0.7)	1365 (0.3)	25 (0.4)	15 (0.4)
Adult personality	7215 (0.9)	250 (0.6)	35 (0.4)	2920 (6.7)	535 (0.4)	1095 (1.0)	135 (2.5)	2170 (0.4)	45 (0.8)	30 (1.0)

Notes. Cells with a sample size of five or less and cells in which a value presented could result in the calculation of a sample size of less than 5 in a complementary cell are presented as <10. Disorder classes are not mutually exclusive. TBI = traumatic brain injury.
[a]Indicates multiple traumas on the day of the first-recorded trauma. [b]Behavioral syndromes associated with physiological disturbances and physical factors (e.g., eating disorders).

© 2024 [First author last name and initials or group name] et al. *[Journal name].*

eFigure 1. SMRs for Psychiatric Disorder Classes Within 5 Years of First-Recorded Traumatic Event in Individuals With Pretrauma Psychopathology

© 2024 [First author last name and initials or group name] et al. *[Journal name].*

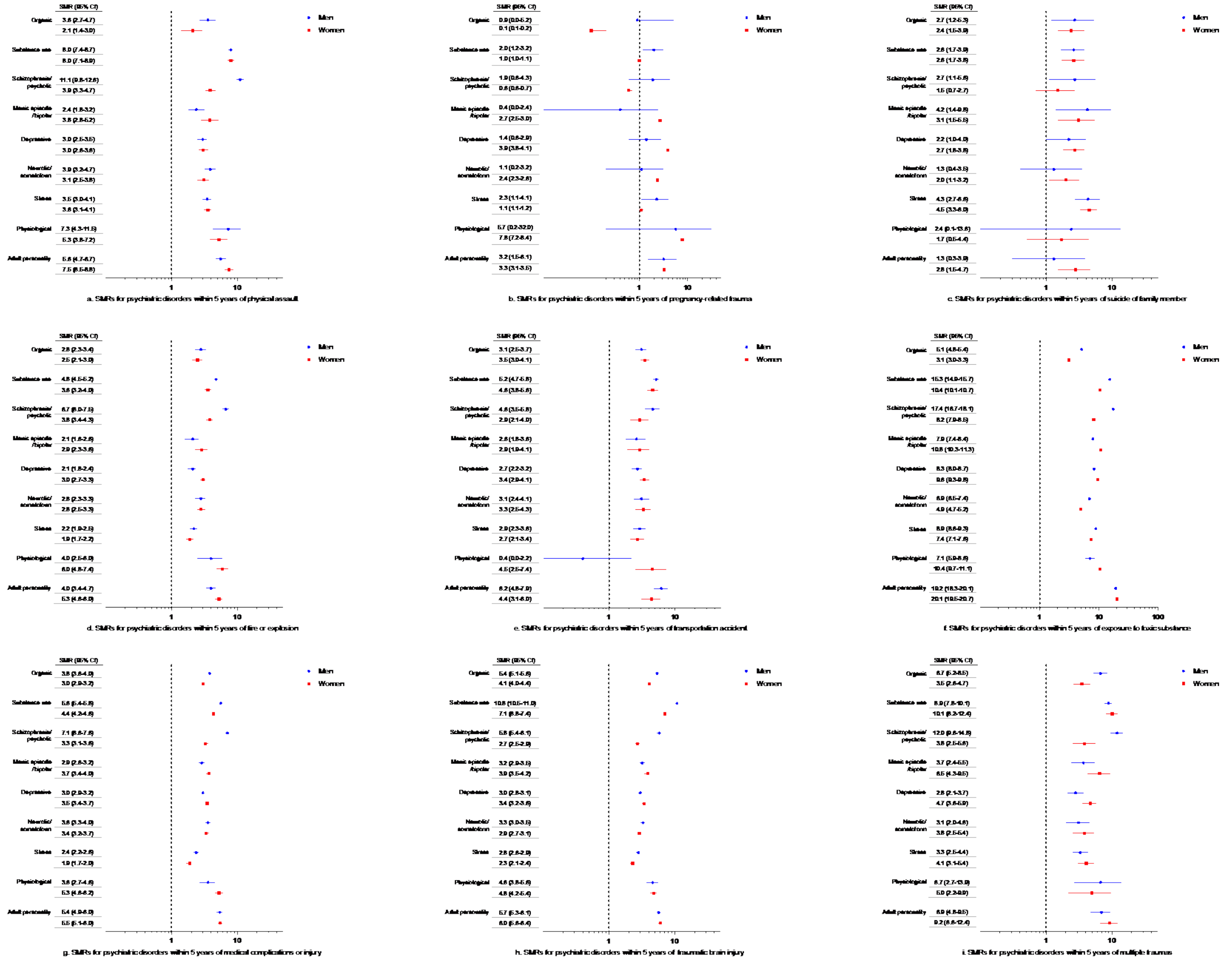

Notes. Interpersonal traumatic events are displayed in panels a-c. Non-interpersonal traumatic events are displayed in panels d-i. Disorder classes are not mutually exclusive. “Physiological” refers to behavioral syndromes associated with physiological disturbances and physical factors (e.g., eating disorders, sleep disorders). CI = confidence interval; SMR = standardized morbidity ratio.

© 2024 [First author last name and initials or group name] et al. *[Journal name].*

eFigure 2. SMRs for Psychiatric Disorder Classes Within 5 Years of First-Recorded Traumatic Event in Individuals Without Pretrauma Psychopathology

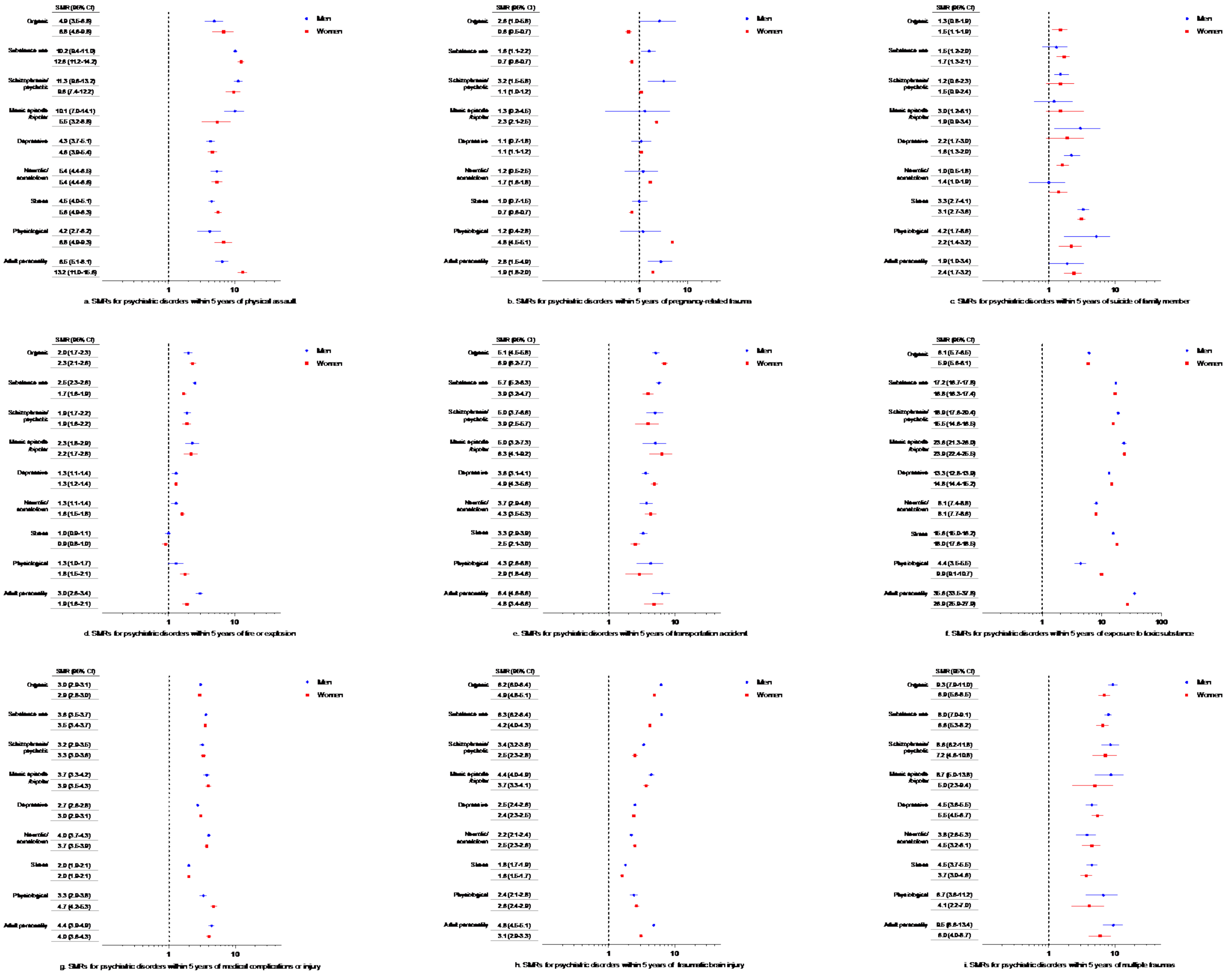

Notes. Interpersonal traumatic events are displayed in panels a-c. Non-interpersonal traumatic events are displayed in panels d-i. Disorder classes are not mutually exclusive. “Physiological” refers to behavioral syndromes associated with physiological disturbances and physical factors (e.g., eating disorders, sleep disorders). CI = confidence interval; SMR = standardized morbidity ratio.

© 2024 [First author last name and initials or group name] et al. *[Journal name].*

eTable 8. Demographic Characteristics of Men and Women in the Danish Health Registry Trauma Cohort With Exposure to Toxic Substance

	Men with exposure to toxic substance in trauma cohort (n=46,450)		Women with exposure to toxic substance in trauma cohort (n=61,765)		Men with exposure and pre-trauma psychopathology (n=13,400)		Women with exposure and pre-trauma psychopathology (n=18,485)		Men with exposure and without pre-trauma psychopathology (n=33,050)		Women with exposure and without pre-trauma psychopathology (n=43,280)	
Variable	Exposure to drugs or medication (n=22,935)	Exposure to toxic substance (n=23,515)	Exposure to drugs or medication (n=23,250)	Exposure to toxic substance (n=38,515)	Exposure to drugs or medication (n=4,030)	Exposure to toxic substance (n=9,370)	Exposure to drugs or medication (n=4,630)	Exposure to toxic substance (n=13,855)	Exposure to drugs or medication (n=18,905)	Exposure to toxic substance (n=14,145)	Exposure to drugs or medication (n=18,620)	Exposure to toxic substance (n=24,660)
	n (%)	n (%)	n (%)	n (%)	n (%)	n (%)	n (%)	n (%)	n (%)	n (%)	n (%)	n (%)
Age (years)												
< 16	4,575 (19.9)	1,790 (7.6)	4,080 (17.5)	5,745 (14.9)	110 (2.8)	180 (1.9)	245 (5.3)	975 (7.0)	4,465 (23.6)	1,610 (11.4)	3,830 (20.6)	4,770 (19.3)
16-39	5,105 (22.3)	9,845 (41.9)	4,795 (20.6)	15,805 (41.0)	1,190 (29.5)	3,725 (39.8)	1,355 (29.2)	5,615 (40.5)	3,915 (20.7)	6,120 (43.3)	3,440 (18.5)	10,190 (41.3)
40-59	5,790 (25.2)	7,130 (30.3)	4,545 (19.5)	8,660 (22.5)	1,525 (37.9)	3,630 (38.8)	1,345 (29.0)	4,020 (29.0)	4,265 (22.6)	3,500 (24.7)	3,200 (17.2)	4,640 (18.8)
≥ 60	7,465 (32.6)	4,750 (20.2)	9,835 (42.3)	8,310 (21.6)	1,205 (29.9)	1,830 (19.6)	1,685 (36.4)	3,245 (23.4)	6,260 (33.1)	2,915 (20.6)	8,150 (43.8)	5,065 (20.5)
Income quartile												
<Q1 (lowest)	3,600 (15.7)	5,900 (25.1)	5,155 (22.2)	14,510 (37.7)	840 (20.8)	2,310 (24.7)	1,260 (27.2)	4,895 (35.3)	2,765 (14.6)	3,590 (25.4)	3,895 (20.9)	9,620 (39.0)
Q1 - < Q2	6,240 (27.2)	7,955 (33.8)	8,255 (35.5)	11,150 (28.9)	1,795 (44.5)	3,950 (42.2)	1,935 (41.8)	4,945 (35.7)	4,445 (23.5)	4,005 (28.3)	6,320 (33.9)	6,205 (25.2)
Q2 - < Q3	3,830 (16.7)	4,410 (18.8)	4,060 (17.5)	6,555 (17.0)	795 (19.7)	1,855 (19.8)	920 (19.9)	2,585 (18.7)	3,035 (16.1)	2,555 (18.1)	3,140 (16.9)	3,965 (16.1)
≥Q3 (highest)	4,770 (20.8)	3,595 (15.3)	2,060 (8.9)	2,725 (7.1)	500 (12.4)	1,085 (11.6)	370 (8.0)	935 (6.7)	4,270 (22.6)	2,505 (17.7)	1,690 (9.1)	1,790 (7.3)
Child[a]	4,300 (18.7)	1,295 (5.5)	3,270 (14.1)	2,100 (5.4)	70 (1.8)	65 (0.7)	80 (1.8)	195 (1.4)	4,230 (22.4)	1,225 (8.7)	3,185 (17.1)	1,900 (7.7)
Missing	195 (0.9)	365 (1.5)	450 (1.9)	1,480 (3.8)	35 (0.9)	100 (1.1)	60 (1.3)	295 (2.1)	160 (0.8)	260 (1.9)	390 (2.1)	1,180 (4.8)
Marital status												
Married/ registered partner	8,420 (36.7)	6,115 (26.0)	6,330 (27.2)	8,570 (22.3)	1,145 (28.4)	2,050 (21.9)	1,125 (24.3)	3,075 (22.2)	7,275 (38.5)	4,070 (28.8)	5,210 (28.0)	5,495 (22.3)
Single	10,255 (44.7)	12,680 (53.9)	8,585 (36.9)	20,540 (53.3)	1,810 (44.9)	5,075 (54.2)	1,710 (36.9)	6,800 (49.1)	8,445 (44.7)	7,605 (53.7)	6,875 (36.9)	13,740 (55.7)
Divorced	2,600 (11.3)	3,510 (14.9)	3,010 (12.9)	5,380 (14.0)	820 (20.4)	1,885 (20.1)	930 (20.1)	2,645 (19.1)	1,780 (9.4)	1,620 (11.5)	2,080 (11.2)	2,735 (11.1)
Widowed	1,525 (6.6)	1,120 (4.8)	5,190 (22.3)	3,915 (10.2)	250 (6.2)	340 (3.6)	865 (18.6)	1,315 (9.5)	1,270 (6.7)	780 (5.5)	4,330 (23.3)	2,600 (10.5)
Unknown	140 (0.6)	95 (0.4)	135 (0.6)	110 (0.3)	<10	20 (0.2)	<10	20 (0.1)	135 (0.7)	75 (0.5)	130 (0.7)	90 (0.4)

Notes. Cells with a sample size of five or less and cells in which a value presented could result in the calculation of a sample size of less than 5 in a complementary cell are presented as <10.
[a]Persons under the age of 14 with no income data.

© 2024 [First author last name and initials or group name] et al. *[Journal name].*

eTable 9. Incidence of Psychiatric Disorders Within 5 Years Following First-Recorded Exposure to Toxic Substance in Men and Women

	Exposure to toxic substance in trauma cohort (n=108,215)		Exposure to toxic substance and pre-trauma psychopathology (n=31,885)		Exposure to toxic substance and without pre-trauma psychopathology (n=76,330)	
Men	Exposure to drugs or medication (n=22,935)	Exposure to toxic substance (n=23,515)	Exposure to drugs or medication (n=4,030)	Exposure to toxic substance (n=9,370)	Exposure to drugs or medication (n=18,905)	Exposure to toxic substance (n=14,145)
	n (%)	n (%)	n (%)	n (%)	n (%)	n (%)
None	17645 (76.9)	10995 (46.7)	1445 (35.9)	2500 (26.7)	16195 (85.7)	8495 (60.0)
Organic	920 (4.0)	1210 (5.1)	400 (9.9)	715 (7.6)	525 (2.8)	495 (3.5)
Substance Use	2910 (12.7)	7110 (30.2)	1665 (41.3)	4445 (47.4)	1245 (6.6)	2665 (18.8)
Schizophrenia/ psychotic	765 (3.3)	2310 (9.8)	570 (14.2)	1785 (19.1)	195 (1.0)	525 (3.7)
Manic episode/bipolar	375 (1.6)	1040 (4.4)	260 (6.5)	745 (8.0)	110 (0.6)	295 (2.1)
Depressive	1300 (5.7)	3410 (14.5)	635 (15.7)	1820 (19.4)	665 (3.5)	1590 (11.2)
Neurotic/somatoform	370 (1.6)	1050 (4.5)	225 (5.5)	670 (7.1)	145 (0.8)	380 (2.7)
Stress	1220 (5.3)	3665 (15.6)	525 (13.0)	1560 (16.7)	695 (3.7)	2105 (14.9)
Physiological[a]	70 (0.3)	125 (0.5)	35 (0.8)	80 (0.9)	35 (0.2)	45 (0.3)
Adult personality	690 (3.0)	2085 (8.9)	415 (10.3)	1310 (14.0)	275 (1.4)	775 (5.5)
Women	Exposure to drugs or medication (n=23,250)	Exposure to toxic substance (n=38,515)	Exposure to drugs or medication (n=4,630)	Exposure to toxic substance (n=13,855)	Exposure to drugs or medication (n=18,620)	Exposure to toxic substance (n=24,660)
	n (%)	n (%)	n (%)	n (%)	n (%)	n (%)
None	16345 (70.3)	17175 (44.6)	1570 (33.9)	3300 (23.8)	14780 (79.4)	13875 (56.3)
Organic	1350 (5.8)	1705 (4.4)	475 (10.2)	915 (6.6)	875 (4.7)	790 (3.2)
Substance Use	2105 (9.1)	6465 (16.8)	1135 (24.5)	3820 (27.6)	970 (5.2)	2645 (10.7)
Schizophrenia/ psychotic	930 (4.0)	3130 (8.1)	650 (14.0)	2340 (16.9)	280 (1.5)	795 (3.2)
Manic episode/bipolar	710 (3.0)	2255 (5.9)	460 (10.0)	1585 (11.4)	245 (1.3)	670 (2.7)
Depressive	2325 (10.0)	8025 (20.8)	1100 (23.8)	4095 (29.5)	1225 (6.6)	3930 (15.9)
Neurotic/somatoform	750 (3.2)	2590 (6.7)	420 (9.0)	1565 (11.3)	330 (1.8)	1025 (4.2)
Stress	1940 (8.3)	8110 (21.1)	750 (16.2)	3145 (22.7)	1185 (6.4)	4965 (20.1)
Physiological[a]	310 (1.3)	1165 (3.0)	185 (4.0)	720 (5.2)	120 (0.7)	450 (1.8)
Adult personality	1495 (6.4)	5680 (14.7)	870 (18.8)	3400 (24.5)	625 (3.4)	2280 (9.2)

Notes. Disorder classes are not mutually exclusive.

© 2024 [First author last name and initials or group name] et al. *[Journal name].*

[a]Behavioral syndromes associated with physiological disturbances and physical factors (e.g., eating disorders).

eTable 10. Standardized Morbidity Ratio (SMR) for Psychiatric Disorder Classes Within 5 Years of First-Recorded Exposure to Toxic Substance in Men and Women

	Exposure to toxic substance in trauma cohort (n=108,215)		Exposure to toxic substance and pre-trauma psychopathology (n=31,885)		Exposure to toxic substance and without pre-trauma psychopathology (n=76,330)	
Men	Exposure to drugs or medication (n=22,935)	Exposure to toxic substance (n=23,515)	Exposure to drugs or medication (n=4,030)	Exposure to toxic substance (n=9,370)	Exposure to drugs or medication (n=18,905)	Exposure to toxic substance (n=14,145)
	SMR (95% CI)	SMR (95% CI)	SMR (95% CI)	SMR (95% CI)	SMR (95% CI)	SMR (95% CI)
None	5.8 (5.5-6.2)	14.4 (13.6-15.2)	5.2 (4.7-5.7)	5.1 (4.8-5.5)	4.4 (4.1-4.8)	10.3 (9.4-11.3)
Organic	13.9 (13.4-14.4)	47.5 (46.4-48.6)	13.4 (12.8-14.1)	16.1 (15.6-16.6)	8.5 (8.0-8.9)	33.3 (32.1-34.6)
Substance Use	20.0 (18.6-21.5)	70.8 (67.9-73.7)	14.7 (13.6-16.0)	18.4 (17.6-19.3)	8.1 (7.0-9.4)	37.2 (34.1-40.5)
Schizophrenia/ psychotic	16.7 (15.0-18.4)	51.2 (48.2-54.5)	7.0 (6.1-7.9)	8.3 (7.8-9.0)	10.3 (8.5-12.4)	45.9 (40.8-51.4)
Manic episode/bipolar	8.5 (8.0-9.0)	31.1 (30.1-32.2)	6.8 (6.3-7.3)	9.1 (8.7-9.5)	6.0 (5.5-6.5)	27.2 (25.9-28.6)
Depressive	6.2 (5.6-6.9)	22.8 (21.4-24.2)	6.1 (5.4-7.0)	7.3 (6.7-7.9)	3.5 (2.9-4.1)	16.4 (14.8-18.2)
Neurotic/somatoform	8.3 (7.8-8.8)	32.0 (30.9-33.0)	7.9 (7.2-8.6)	9.4 (8.9-9.9)	6.1 (5.6-6.5)	32.7 (31.3-34.1)
Stress	5.0 (3.9-6.3)	9.2 (7.7-11.0)	7.1 (4.9-10.0)	7.2 (5.7-8.9)	3.5 (2.5-4.9)	5.6 (4.0-7.5)
Physiological[a]	25.3 (23.4-27.2)	80.3 (76.9-83.9)	17.4 (15.8-19.2)	19.8 (18.8-20.9)	16.0 (14.2-18.0)	62.4 (58.1-66.9)
Adult personality	5.8 (5.5-6.2)	14.4 (13.6-15.2)	5.2 (4.7-5.7)	5.1 (4.8-5.5)	4.4 (4.1-4.8)	10.3 (9.4-11.3)
Women	Exposure to drugs or medication (n=23,250)	Exposure to toxic substance (n=38,515)	Exposure to drugs or medication (n=4,630)	Exposure to toxic substance (n=13,855)	Exposure to drugs or medication (n=18,620)	Exposure to toxic substance (n=24,660)
	SMR (95% CI)	SMR (95% CI)	SMR (95% CI)	SMR (95% CI)	SMR (95% CI)	SMR (95% CI)
None	5.6 (5.3-5.9)	8.7 (8.3-9.1)	3.5 (3.2-3.8)	2.9 (2.7-3.1)	5.0 (4.7-5.3)	7.3 (6.8-7.8)
Organic	15.3 (14.6-15.9)	27.4 (26.8-28.1)	10.0 (9.4-10.6)	10.5 (10.2-10.9)	10.9 (10.2-11.6)	21.0 (20.2-21.8)
Substance Use	15.9 (14.9-16.9)	28.7 (27.7-29.7)	7.6 (7.0-8.2)	8.4 (8.0-8.7)	10.4 (9.3-11.7)	18.7 (17.5-20.1)
Schizophrenia/ psychotic	20.3 (18.9-21.9)	42.1 (40.4-43.9)	9.4 (8.5-10.3)	11.3 (10.8-11.9)	14.8 (13.0-16.8)	31.0 (28.7-33.4)
Manic episode/bipolar	9.9 (9.5-10.3)	25.9 (25.4-26.5)	7.6 (7.2-8.1)	10.3 (10.0-10.6)	7.4 (7.0-7.8)	21.5 (20.9-22.2)
Depressive	7.2 (6.7-7.7)	13.3 (12.8-13.8)	4.7 (4.2-5.1)	5.0 (4.8-5.3)	5.1 (4.5-5.7)	10.1 (9.5-10.7)
Neurotic/somatoform	9.4 (9.0-9.8)	24.3 (23.8-24.9)	6.1 (5.7-6.5)	7.8 (7.5-8.1)	8.1 (7.6-8.5)	25.6 (24.9-26.4)
Stress	9.7 (8.7-10.9)	19.6 (18.5-20.7)	10.9 (9.4-12.6)	10.3 (9.6-11.1)	5.5 (4.6-6.6)	12.5 (11.4-13.8)
Physiological[a]	24.9 (23.6-26.2)	46.8 (45.6-48.0)	19.5 (18.2-20.8)	20.3 (19.6-21.0)	16.3 (15.1-17.7)	32.6 (31.3-34.0)
Adult personality	5.6 (5.3-5.9)	8.7 (8.3-9.1)	3.5 (3.2-3.8)	2.9 (2.7-3.1)	5.0 (4.7-5.3)	7.3 (6.8-7.8)

© 2024 [First author last name and initials or group name] et al. *[Journal name].*

Notes. Disorder classes are not mutually exclusive.
[a]Behavioral syndromes associated with physiological disturbances and physical factors (e.g., eating disorders).

© 2024 [First author last name and initials or group name] et al. *[Journal name].*

eReferences

1. Schmidt, M., et al., The Danish health care system and epidemiological research: From health care contacts to database records. Clinical Epidemiology, 2019: p. 563-591.
2. Gaist D, Væth M, Tsiropoulos I, Christensen K, et al. Risk of subarachnoid haemorrhage in first degree relatives of patients with subarachnoid haemorrhage: follow up study based on national registries in Denmark. BMJ, 2000. 320(7228): p. 141-145.
   Schmidt M, Pedersen L, Sørensen HT. The Danish Civil Registration System as a tool in epidemiology. European Journal of Epidemiology, 2014. 29: p. 541-549.
3. Baadsgaard M, Quitzau J. Danish registers on personal income and transfer payments. Scandinavian Journal of Public Health, 2011. 39(7_suppl): p. 103-105.
4. World Health Organization. International Statistical Classification of Diseases and related health problems: Alphabetical index. Vol. 3. 2004: World Health Organization.
5. Mors O, Perto GP, Mortensen PB. The Danish psychiatric central research register. Scandinavian Journal of Public Health, 2011. 39(7_suppl): p. 54-57.
6. Svensson E, Lash TL, Resick PA, et al., Validity of reaction to severe stress and adjustment disorder diagnoses in the Danish Psychiatric Central Research Registry. Clinical Epidemiology, 2015: p. 235-242.
7. Schmidt M, Schmidt SA, Sandegaard JL, et al. The Danish National Patient Registry: a review of content, data quality, and research potential. Clinical Epidemiology, 2015: p. 449-490.
8. Bliddal M, Broe A, Pottegård A, et al. The Danish medical birth register. European Journal of Epidemiology, 2018. 33: p. 27-36.
9. Klemmensen ÅK, Olsen SF, Østerdal ML, et al. Validity of preeclampsia-related diagnoses recorded in a national hospital registry and in a postpartum interview of the women. American Journal of Epidemiology, 2007. 166(2): p. 117-124.
10. Sneider K, Langhoff-Roos J, Sundtoft IB, et al. Validation of second trimester miscarriages and spontaneous deliveries. Clinical Epidemiology, 2015: p. 517-527.
11. Thisted DL, Mortensen LH, Hvidman L, et al. Use of ICD-10 codes to monitor uterine rupture: validation of a national birth registry. European Journal of Obstetrics & Gynecology and Reproductive Biology, 2014. 173: p. 23-28.
12. Helweg-Larsen K. The Danish register of causes of death. Scandinavian Journal of Public Health, 2011. 39(7_suppl): p. 26-29.
13. Tøllefsen IM, Helweg-Larsen K, Thiblin I, et al. Are suicide deaths under-reported? Nationwide re-evaluations of 1800 deaths in Scandinavia. BMJ Open, 2015. 5(11): p. e009120.
14. Gradus JL, Rosellini AJ, Szentkúti P, et al. Using Danish national registry data to understand psychopathology following potentially traumatic experiences. *J Trauma Stress*. 2022;35(2):619-630.
15. Rothman K. Epidemiologic analysis with a programmable calculator. 1979: US Department of Health, Education, and Welfare, Public Health Service, National Institutes of Health.
16. Breslow NE, Day NE. Statistical methods in cancer research II. The design and analysis of cohort studies. IARC Scientific Publish, 1987. 82: p. 1-406.

© 2024 [First author last name and initials or group name] et al. *[Journal name].*
