# Supplementary material for: Sex Differences in Psychopathology Following Potentially Traumatic Experiences
Source: JAMA Netw Open. 2024 Feb 22;7(2):e240201. doi: 10.1001/jamanetworkopen.2024.0201 (PMC10884878; doi:10.1001/jamanetworkopen.2024.0201)
Supplement: Supplement 2. — Data Sharing Statement [file jamanetwopen-e240201-s002.pdf]

Data Sharing Statement

Data
Data available: No

Additional Information
Explanation for why data not available: These data will not be shared due to their sensitive nature but are available to qualified investigators for re-analysis upon reasonable request.
